# Unlocking Implantation: The Role of Nitric Oxide, NO_2_-NO_3_, and eNOS in Endometrial Receptivity and IVF Success—A Systematic Review

**DOI:** 10.3390/ijms26146569

**Published:** 2025-07-08

**Authors:** Charalampos Voros, Iwakeim Sapantzoglou, Despoina Mavrogianni, Diamantis Athanasiou, Antonia Varthaliti, Kyriakos Bananis, Antonia Athanasiou, Aikaterini Athanasiou, Anthi-Maria Papahliou, Constantinos G. Zografos, Athanasios Gkirgkinoudis, Ioannis Papapanagiotou, Kyriaki Migklis, Dimitris Mazis Kourakos, Georgios Papadimas, Maria Anastasia Daskalaki, Panagiotis Antsaklis, Dimitrios Loutradis, Georgios Daskalakis

**Affiliations:** 1 1st Department of Obstetrics and Gynecology, ‘Alexandra’ General Hospital, National and Kapodistrian University of Athens, 80 VasilissisSofias Avenue, 11528 Athens, Greece; kimsap1990@hotmail.com (I.S.); depy.mavrogianni@yahoo.com (D.M.); antonia.varthaliti@hotmail.com (A.V.); anthipapahliou@gmail.com (A.-M.P.); tgkirgki@gmail.com (A.G.); md181341@students.euc.ac.cy (M.A.D.); panosant@gmail.com (P.A.); 2 IVF Athens Reproduction Center V. Athanasiou, 15123 Maroussi, Greece; diamathan16@gmail.com (D.A.); antoathan16@gmail.com (A.A.); diamathan17@gmail.com (A.A.); 3 King’s College Hospitals NHS Foundation Trust, London SE5 9RS, UK; kyriakos.bananis@nhs.net; 4 2nd Surgical Department, General Hospital of Athens “LAIKO”, 11527 Athens, Greece; koszogra92@hotmail.com; 5 Athens Medical School, National and Kapodistrian University of Athens, 15772 Athens, Greece; gpapamd@hotmail.com (I.P.); kyriaki.migklis@gmail.com (K.M.); mazisdimitris@gmail.com (D.M.K.); dr.georgepapadimas@gmail.com (G.P.); loutradi@otenet.gr (D.L.); 6 Fertility Institute-Assisted Reproduction Unit, Paster 15, 11528 Athens, Greece

**Keywords:** nitric oxide, NO_2_^−^, NO_3_^−^, eNOS, endometrial receptivity, NOS3 polymorphisms, implantation failure, in vitro fertilization (IVF), recurrent pregnancy loss, unexplained infertility, endometrial microenvironment

## Abstract

Nitric oxide (NO) predominantly regulates endometrial receptivity, angiogenesis, immunological tolerance, and trophoblast invasion throughout the implantation period. Both insufficient and excessive nitric oxide production have been linked to suboptimal embryo implantation and infertility. The primary enzymatic source of uterine nitric oxide, along with hormonal, metabolic, and immunological variables and genetic variations in the endothelial nitric oxide synthase gene (NOS3), affects endothelial nitric oxide synthase (eNOS). Despite its considerable importance, there is limited knowledge regarding the practical implementation of nitric oxide-related diagnoses and therapies in reproductive medicine. A comprehensive assessment was performed in accordance with the PRISMA principles. Electronic searches were carried out in PubMed, Scopus, and Embase, and we analyzed the literature published from 2000 to 2024 regarding the association between NO, its metabolites (NO_2_^−^ and NO_3_^−^), eNOS expression, NOS3 gene variants, and reproductive outcomes. Relevant studies encompassed clinical trials, observational studies, and experimental research using either human or animal subjects. We collected data about therapeutic interventions, hormonal and immunological associations, nitric oxide measurement techniques, and in vitro fertilization success rates. A total of thirty-four studies were included. Dysregulated nitric oxide signaling, characterized by modified eNOS expression, oxidative stress, or NOS3 polymorphisms (e.g., Glu298Asp and intron 4 VNTR), was linked to diminished endometrial receptivity and an elevated risk of implantation failure and miscarriage. The dynamics of local uterine NO are essential as elevated and diminished systemic levels of NO_2_^−^/NO_3_^−^ corresponded with enhanced and decreased implantation rates, respectively. Among many therapeutic approaches, targeted hormone treatments, antioxidant therapy, and dietary nitrate supplements have demonstrated potential in restoring nitric oxide balance and enhancing reproductive outcomes. In animal models, the modification of nitric oxide significantly impacted decidualization, angiogenesis, and embryo viability. Nitric oxide is a multifaceted molecular mediator with considerable ramifications for successful implantation. Its therapeutic and diagnostic efficacy increases with its sensitivity to environmental, hormonal, and genetic alterations. Integrating targeted nitric oxide modulation, oxidative stress assessment, and NOS3 genotyping with personalized reproductive therapy will enhance endometrial receptivity and improve IVF outcomes. Future translational research should incorporate nitric oxide signaling into personalized treatment protocols for patients with unexplained infertility or recurrent implantation failure.

## 1. Introduction

The process of embryo implantation in human reproduction is intricate and meticulously regulated. Precise molecular communication is required between a blastocyst prepared for development and an endometrium prepared for reception [1]. Usually spanning days 20–24 of a normal 28-day menstrual cycle, this bidirectional molecular interaction takes place inside a small temporal window called the window of implantation (WOI) [2]. The uterine lining changes extensively in structural, biochemical, and immunological manners during this phase to enable blastocyst apposition, adhesion, and later trophoblast invasion. Even with the amazing advancement in ART, especially IVF, implantation failure still presents a major barrier to reaching healthy pregnancies [3]. Although high-quality embryos can be produced and transmitted with ever-increasing accuracy, clinical pregnancy rates per embryo transfer remain unsatisfactory, mostly because of endometrial receptivity rather than embryo quality [4]. Frequent reflection of an underlying disorder in the mother environment in repeated implantation failure (RIF), inexplicable infertility (UI), and early pregnancy loss (EPL) emphasizes the necessity for greater knowledge of the molecular regulators of implantation [5].

Among the several signaling pathways linked to implantation, vascular, inflammatory, and oxidative processes are thought to be crucial. NO, a gaseous signaling molecule that affects vasodilation, leukocyte recruitment, cytokine signaling, and stromal–epithelial communication, is crucial to these mechanisms [6]. More and more individuals are discovering that being able to manage the generation, availability, and downstream signaling of NO is very important for successful implantation. This is largely because NO affects endometrial perfusion, vascular permeability, immunological tolerance, and angiogenesis, all of which are necessary for making the endometrium a good site for an early embryo to grow [3].

NO is endogenously synthesized by the enzymatic action of NOS, which convert L-arginine into L-citrulline, producing NO as a byproduct [7]. Among the three identified isoforms—neuronal (nNOS or NOS1), inducible (iNOS or NOS2), and endothelial (eNOS or NOS3)—eNOS is particularly significant in reproductive physiology [8]. It is consistently expressed on the luminal surface of the endometrium, glandular epithelium, and blood endothelium; its expression is regulated by estrogen and progesterone levels throughout the menstrual cycle [9]. The maximum expression of eNOS indicates that it has a physiological role in priming the endometrium for embryo adhesion and invasion, aligning with the peri-implantation period.

At the molecular level, NO produced by eNOS catalyzes the transformation of GTP into cGMP by activating sGC in adjacent smooth muscle and stromal cells [10]. The prerequisites for an optimal implantation environment include vascular smooth muscle relaxation, increased uterine blood flow, and enhanced endometrial perfusion, all of which are a consequence of this second messenger [11]. NO signaling interacts with downstream targets involved in ECM remodeling, such as integrins and MMPs, hence facilitating trophoblast invasion and spiral artery remodeling [12].

In addition to its role in circulation, nitric oxide regulates immunity at the feto-maternal interface. Minimal levels of nitric oxide promote immunological tolerance, decrease leukocyte adhesion, and facilitate the differentiation of macrophages into M2 (anti-inflammatory) phenotypes—processes essential for preventing maternal immune rejection of the semi-allogeneic embryo [13].

Dysregulated or excessive nitric oxide synthesis, particularly from iNOS, may lead to nitrosative stress, tissue damage, and mortality, all of which are associated with RPL and implantation failure [14]. Moreover, recent research by Melford et al. suggests that NO may engage with the ECS, a novel regulator of implantation competence [15]. In vitro findings indicated that in receptive endometrial epithelial cells, nitric oxide donors (e.g., SNAP) elevated the expression of fatty acid amide hydrolase (FAAH) and diminished N-acylphosphatidylethanolamine phospholipase D (NAPE-PLD) [15]. These alterations reduce the concentration of anandamide (AEA), an endocannabinoid whose accumulation impairs implantation, hence enhancing the molecular characteristic of receptivity.

A variety of reproductive disorders characterized by implantation failure or early pregnancy loss has been associated with alterations in nitric oxide production or endothelial nitric oxide synthase expression [16]. One disease is endometriosis-associated infertility, characterized by the eutopic endometrium—despite being histologically normal—exhibiting persistently elevated eNOS levels during the menstrual cycle [17]. Excessive nitric oxide production due to this aberrant overexpression may generate oxidative stress, disrupt the endometrial redox equilibrium, and interfere with the pro-implantation gene network. Women with stage III–IV endometriosis had significantly elevated eNOS levels in a rigorously controlled research study by Wang et al. compared to fertile controls [18]. Subsequent to GnRH agonist administration, a notable downregulation of eNOS expression occurred, accompanied by increased endometrial receptivity, thus underscoring the impact of hormonal modulation on the nitric oxide pathway.

Research on women experiencing IRM or unexplained infertility has demonstrated significantly increased eNOS expression, predominantly in the luminal and glandular epithelium, during the peri-implantation phase [19]. Najafi et al. showed that the deregulation of pro-survival and apoptotic signaling pathways, improper decidualization, or nitrosative injury may result in a detrimental endometrial milieu due to such overexpression. These findings indicate a precarious equilibrium: whereas physiological nitric oxide levels are essential for implantation, both deficiency and surplus may adversely affect reproductive outcomes [20].

In addition to studies on protein expression, genetic variations in the NOS3 gene, which encodes eNOS, have been examined for their impact on RPL susceptibility and implantation failure. The 27 bp variable number tandem repeat (VNTR) in intron 4 and the Glu298Asp missense mutation (G894T and rs179998) are among the most extensively studied variants [21]. Some studies, such as that by Banerjee et al., indicated significantly reduced systemic NO levels and downregulated eNOS in women with recurrent pregnancy loss (RPL) [22]. In contrast, other research, including substantial cohorts from Karvela et al., Hefler et al., and Makino et al., did not find a consistent correlation between NOS3 polymorphisms and reproductive failure. These variances may represent ethnic variety, alterations in inclusion criteria, or the influence of epigenetic and environmental modifications on gene expression [23,24,25].

Furthermore, attempts to utilize systemic NO metabolites (NO_2_^−^) as proxy indicators of endometrial function have yielded contradictory findings. Fábregues et al. observed that IVF patients exhibited no significant differences in serum nitrite/nitrate levels between conception and non-conception cycles. This suggests that assessing endometrial receptivity would benefit more from localized tissue-specific nitric oxide activity than from systemic levels [26].

Significant information gaps persist despite the growing body of data connecting nitric oxide signaling to endometrial physiology and embryo implantation [27]. Published research varies significantly regarding study design, population characteristics, the cycle phase of endometrial collection, methods of nitric oxide quantification, and definitions of clinical objectives [28]. Moreover, whereas certain studies indicate a harmful role of aberrant eNOS expression or NOS3 polymorphisms in infertility and miscarriage, others present neutral or conflicting findings, hence limiting the generalizability of the data [29]. Therapeutic strategies targeting the NO pathway have not been widely implemented in ART procedures, nor has the therapeutic significance of monitoring NO_2_^−^/NO_3_^−^ levels or assessing eNOS expression as diagnostic biomarkers of receptivity been conclusively demonstrated.

A systematic and critical examination of the existing literature is warranted to consolidate current material and identify coherent molecular patterns or clinical associations. This thorough study aims to evaluate and integrate the current understanding of the role of NO, its stable metabolites NO_2_^−^ and NO_3_^−^, and eNOS in endometrial receptivity and IVF outcomes. Particular emphasis is placed on the underlying molecular mechanisms, gene expression studies, polymorphism analyses, and translational implications of modifications to the NO pathway within the context of assisted reproduction.

## 2. Material and Methods

### 2.1. Protocol Reporting and Registration Guidelines

The Preferred Reporting Items for Systematic Reviews and Meta-Analyses (PRISMA) 2020 criteria guided the methodical review that was undertaken in this review. The protocol’s registration number is CRD42025101, which was prospectively recorded in the PROSPERO international prospective register of systematic reviews [30].

### 2.2. Review Question

This review aimed to address the following research question: how does nitric oxide (NO), its stable metabolites (NO_2_^−^ and NO_3_^−^), and eNOS control endometrial receptacle and ascertain the results of IVF?

### 2.3. Eligibility Criteria

Studies investigating the role of NO, its stable oxidative metabolites—specifically NO_2_^−^ and NO_3_^−^—and eNOS, encoded by the NOS3 gene concerning critical reproductive outcomes, such as endometrial receptivity, embryo implantation, and the efficacy of IVF, were considered suitable for inclusion in this systematic review. Eligible research included human participants and original data sourced from clinical cohorts or molecular investigations utilizing human tissues, including but not limited to blood samples, follicular fluid, or endometrial biopsies collected during the peri-implantation window, as is shown in Table 1. This review incorporated a diverse range of research methodologies to align with the multidisciplinary nature of the topic. We examined prospective and retrospective cohort studies, RCTs, and case–control studies that investigated genomic, genetic, biochemical, or clinical parameters associated with nitric oxide signaling. Additionally, acceptable studies included experimental research mimicking the implantation window through human-derived tissue explants, as well as verified in vitro models demonstrating the expression, regulation, or functional involvement of eNOS or nitric oxide-related pathways utilizing primary human endometrial epithelial or stromal cells.

Relevant studies included those that recorded at least one reproductive outcome directly associated with implantation potential—such as implantation rate, clinical pregnancy rate, biochemical pregnancy, RIF, or RPL—to ensure therapeutic significance. Additionally, studies evaluating systemic NO metabolite levels in connection with IVF or natural conception results were eligible, provided they had quantitative assessments and group comparison analyses.

Research utilizing cell lines not derived from human endometrial or reproductive tissues, or conducted exclusively in non-human animal models, was excluded. Theoretical discussions lacking empirical evidence, mathematical modeling articles, and silica simulations were also excluded. Narrative reviews, systematic reviews, meta-analyses, expert opinions, editorials, letters to the editor, and case studies were not included. Studies without sufficient methodological detail or clarity of outcomes were excluded to ensure the integrity of data synthesis.

Additionally, we rejected studies on NO or eNOS in reproductive contexts not pertinent to implantation, namely those focusing solely on oocyte maturation, spermatogenesis, or systemic vascular conditions during pregnancy (e.g., preeclampsia), unless they explicitly connected to endometrial function or IVF success. The final analysis removed non-English studies, abstracts only, and inaccessible full texts; only publications published in English and accessible in full text through academic databases were included.

### 2.4. Search Policy and Information Sources

In Table 2, three main scientific databases—PubMed/MEDLINE, Scopus, and Web of Science—were comprehensively searched in order to find qualified papers released from database inception to 31 March 2024. The search strategy was developed by combining controlled vocabulary (MeSH terms) with free-text keywords related to NO, eNOS, NO_2_^−^/NO_3_^−^ metabolites, endometrial receptacle, implantation, and ART, including IVF. Sensitivity and specificity were maximized using truncation symbols and AND/OR Boolean operators.

Apart from the database searches, the reference lists of the included papers were personally checked to find pertinent studies missed during the main search. Key study citation tracking was also performed with Google Scholar’s “cited by” tool and Web of Science. We only looked at articles published in English available in full-text form. This review excludes studies gleaned from conference abstracts, gray literature, and preprints.

### 2.5. Approach of Study Selection

All found references were imported into Zotero, a reference management tool, after database searches were finished. Using the de-duplication tool of the program, duplicate records were found; two reviewers personally validated and eliminated them to guarantee accuracy. The remaining unusual records underwent a two-phase screening process: first title and abstract screening and then a full-text examination for potentially qualified papers.

Two independent reviewers—C.V. and D.L.—reviewed the titles and abstracts of every obtained reference in the first screening phase to ascertain whether each record satisfied the defined inclusion criteria based on the PICOS framework. At this point, studies absolutely unrelated to the population of interest or the study issue were deleted. The full-text article was obtained for additional review where titles and abstracts lacked enough detail to evaluate eligibility. Full-text versions of all possibly relevant studies were obtained in the second phase and independently evaluated for inclusion by the same two reviewers. Closely examining full texts helped to verify adherence to all eligibility criteria—study design, population, outcome relevance, and NO-related assessment included. Differences in research eligibility were settled by consensus and conversation.

**Figure 1:**
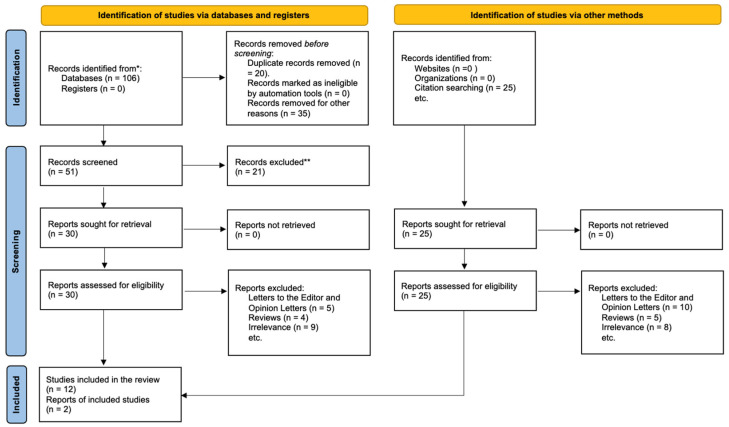
The PRISMA 2020 flow diagram. A flowchart showing the procedure involving the identification, screening, eligibility evaluation, and systematic review of studies following the PRISMA 2020 recommendations [30]. The graph shows the total number of records obtained from databases, duplicates eliminated, records screened, full-text articles evaluated, and the last count of studies incorporated into the qualitative synthesis. There are records of the causes behind excluding full-text articles. * Consider, if feasible to do so, reporting the number of records identified from each database or register searched (rather than the total number across all databases/registers). ** If automation tools were used, indicate how many records were excluded by a human and how many were excluded by automation tools.

Reasons for excluding full-text publications were recorded and classified into particular domains (e.g., irrelevant outcome, non-human study, non-original data, or poor reporting), thereby guaranteeing openness. The process and outcomes of the study selection are shown in a PRISMA 2020 flow diagram (Figure 1), which summarizes the number of records identified through databases and other sources, duplicates eliminated, records screened, full-text reports evaluated for eligibility, reasons for exclusion, and final studies included in the qualitative synthesis.

### 2.6. Data Retraction

To guarantee methodological consistency and repeatability for this review, a standardized and pilot-tested data extraction form was established. Independently applying the form to every qualified study, two reviewers manually extracted all pertinent data. After the extracted data were then checked for consistency, any differences were discussed; in cases when consensus could not be reached, a third reviewer served as adjudicator.

In addition to study design and setting—involving distinguishing whether the study was observational or interventional, prospective or retroactive, and whether it included in vivo or in vitro components—the data extraction process concentrated on gathering comprehensive bibliographic information including the first author, year of publication, and country of origin. We carefully recorded population traits, including diagnosis group definitions (e.g., recurrent pregnancy loss, unexplained infertility, and repeated implantation failure), fertility status, age range, inclusion and exclusion criteria, and, when available, body mass index (BMI). The sample size and the recruiting period were recordable for both cases and controls.

Particularly careful consideration was given to the kind of biological material examined—endometrial biopsy specimens, serum, follicular fluid, or cultured human endometrial epithelial cells. Immunohistochemistry, real-time quantitative PCR, Western blotting, ELISA, and tests for plasma or tissue concentrations of NO_2_^−^ and NO_3_^−^ were among the techniques used to evaluate NO activity or associated pathways. Where relevant, genotyping methods and data on NOS3 gene polymorphisms—e.g., Glu298Asp and intron 4 VNTR—were included.

The main recorded results for every trial were implantation rates, clinical pregnancy rates, miscarriage, and molecular expression levels of NO-related targets, including eNOS. Emphasizing the statistical relevance and directionality of reported correlations, the main results were compiled. Funding sources and conflicts of interest were also recorded whenever they were present.

### 2.7. Risk of Bias 

Each included study underwent a rigorous and systematic assessment of bias to evaluate its internal validity and the reliability of its conclusions. Two independent evaluators conducted the assessment; discrepancies were resolved through consensus or third-party adjudication. The study design informed the selection of quality assessment tools. The Newcastle–Ottawa Scale (NOS) was employed in observational research utilizing cohort and case–control methods. This instrument assesses three fundamental domains: the selection of study groups, the comparability of cases and controls (or cohorts), and the identification of either exposure (for case–control studies) or outcome (for cohort studies). Each domain possesses a score, with research capable of attaining a maximum of 9 stars. A score of 7–9 stars indicates a low risk of bias; a score of 5–6 represents a moderate risk; and a score of ≤4 denotes a high risk.

The Cochrane Risk of Bias 2.0 (RoB 2.0) methodology was employed to evaluate the sole randomized controlled trial included in this review. This instrument assesses potential bias across five domains: the randomization procedure, deviations from intended treatments, absent result data, outcome assessment, and selective reporting. Each domain was assigned a rating of “low risk,” “some concerns,” “high risk,” or “low risk,” which informed the overall evaluation. Although unsuitable for formal NOS or RoB 2.0 methodologies, in vitro experimental studies were evaluated qualitatively. The criteria included appropriate control groups, a definitive experimental design, reproducibility, and clarity in reporting. The studies did not provide a formal numerical score; nevertheless, their methodological strengths and limitations were narratively highlighted in the findings and discussion sections.

Table 3 encapsulates the results of the risk of bias assessment for each included study. Most observational studies exhibited moderate to high quality due to appropriate selection criteria and sample size reporting; nevertheless, there was insufficient adjustment for confounding variables. Issues about blinding and outcome reporting emerged in the randomized trial. Despite the limitations of a limited sample size and the absence of replication across systems, in vitro studies typically demonstrated robust internal consistency.

Validated risk of bias methodologies tailored to each study design facilitated the evaluation of the methodological quality of the fourteen included studies. The majority of case–control and cohort studies, as indicated in Table 3, were evaluated as mediocre in quality mostly due to inadequate reporting of participant selection methods and insufficient control of confounding variables. A few studies, such as Karvela, 2008, and Loizidou, 2021, demonstrated minimal risk of bias due to robust matching of patients and controls and adequate outcome evaluation [23,32]. Due to potential bias in outcome evaluation and insufficient blinding, the solitary randomized controlled study (Ohl et al., 2002) was deemed to have “some concerns.” The two in vitro experiments were assessed narratively, being methodologically robust; however, they were limited by a small sample size and the absence of replication in distinct models [36].

### 2.8. Data Integration

A formal quantitative synthesis, or meta-analysis, was deemed inappropriate due to the variability in study designs, populations, biological samples, outcome measures, and methodologies employed throughout the included studies. A qualitative narrative synthesis was performed, organized around significant mechanistic and clinical themes pertinent to the role of nNO, its stable oxidative metabolites (NO_2_^−^ and NO_3_^−^), and eNOS in endometrial receptivity and implantation success.

Research was thematically categorized into four principal domains for enhanced comparison and interpretation: (1) expression patterns of eNOS in the endometrium under both physiological and pathological conditions (e.g., unexplained infertility, recurrent miscarriage, and endometriosis-associated infertility); (2) systemic or localized concentrations of NO_2_^−^ and NO_3_^−^ concerning implantation and IVF outcomes; (3) the correlation between genetic polymorphisms in the NOS3 gene and reproductive failure; and (4) experimental interventions designed to modulate the NO pathway and their impact on implantation competence. Results within each category were aligned based on the consistency of reported outcomes, sample characteristics, and study quality.

Molecular and histological data (e.g., eNOS mRNA/protein levels, immunohistochemical labeling, and plasma NO measurements) about clinical reproductive outcomes such as implantation rate, biochemical pregnancy, clinical pregnancy rate, and miscarriage were integrated. Receptive and non-receptive endometrial models facilitate in vitro mechanistic research based on the direction and magnitude of NO-related molecular alterations. Table 4 presents a concise review of the included research, detailing their methodological characteristics and principal conclusions; Table 3 summarizes the assessment of bias risk. The synthesis aimed to identify common molecular tendencies, highlight disparities, and examine the translational implications of altered NO signaling within the context of assisted reproduction.

## 3. Results

### 3.1. Overview of Results

The conclusive synthesis of this systematic analysis comprised fourteen articles published from 2000 to 2021. The research comprised one randomized controlled trial, eleven observational studies—either case–control or cohort—and two mechanistic in vitro investigations utilizing human endometrial tissues or cell lines. The selected studies investigated the role of NO, its stable oxidative metabolites (NO_2_^−^ and NO_3_^−^), and eNOS concerning endometrial receptivity, implantation success, recurrent RPL, UI, and RIF in women undergoing either natural conception or assisted reproduction.

The research identification, screening, eligibility assessment, and inclusion process is illustrated in Figure 1, in accordance with the PRISMA 2020 flow diagram [30]. The initial database searches yielded a total of 375 records. Following the removal of duplicates, 294 records remained for title and abstract screening. Out of the 51 full-text papers assessed for eligibility, 37 were excluded based on established criteria, which included irrelevance of results, non-human subjects, non-original data, or inadequate reporting. Fourteen papers met all eligibility criteria and were incorporated into the qualitative synthesis.

Table 4 delineates the methodological characteristics of the 14 studies included following the research selection process indicated in Figure 1. The research varied in demographic factors, design, and methodological approach. Frequently juxtaposed with fertile controls, the majority of studies were case–control investigations involving women with recurrent pregnancy loss, unexplained infertility, or repeated implantation failure. Two in vitro mechanistic studies utilizing human endometrial epithelial or stromal cells were included alongside three cohort studies and one randomized controlled trial. The evaluated biological specimens included endometrial biopsies, serum, follicular fluid, and cultured cells. The assessment methods included immunohistochemistry, RT-PCR, ELISA, nitrite/nitrate assays, and genotyping of NOS3 polymorphisms. The focus was on outcomes such as molecular indicators, eNOS expression, systemic NO metabolite levels, implantation rates, clinical pregnancy, and miscarriage rates. Table 4 provides a systematic summary of the population, intervention or exposure, control group, assessment methods, and principal conclusions related to nitric oxide signaling within the context of endometrial receptivity and IVF success.

Table 4 encapsulates the fourteen studies comprising this systematic review; their design, sample size, biological specimens, and methodological approaches exhibit considerable variance. They concur on a shared scientific emphasis: the assessment of nitric oxide signaling—particularly through eNOS expression, NO_2_^−^/NO_3_^−^ concentrations, and NOS3 gene polymorphisms—within the context of endometrial receptivity and implantation efficacy.

In terms of design, the majority of the studies included—*n* = 11—were observational, with a predominance of case–control designs. Typically, these studies compare women with reproductive disorders—such as RPL, UI, and RIF—to fertile women or those with favorable IVF outcomes. While a solitary randomized controlled trial (Ohl et al., 2002) evaluated the clinical efficacy of nitroglycerin patches—a nitric oxide donor—during embryo transfer [36], prospective cohort studies (e.g., Wang et al., 2006; Fábregues et al., 2000) provided longitudinal insights into nitric oxide-related alterations throughout the cycle or in response to treatment [26,34]. Moreover, two in vitro mechanistic studies (Melford et al., 2021; Telfer et al., 1997) examined the molecular and transcriptional regulation of eNOS and its downstream targets utilizing cultured human endometrial cells [15,37].

The research objective influenced the biological materials assessed during the tests. In research examining eNOS protein or mRNA during the peri-implantation period, endometrial biopsies were the predominant tissue utilized. The biopsy was scheduled between 6 and 10 days post-LH surge, typically coinciding with the peak endometrial receptivity period. Additional analyses assessed systemic or local NO_2_^−^/NO_3_^−^ concentrations utilizing serum, follicular fluid, or embryo culture media. Peripheral blood samples were collected for the investigation of NOS3 polymorphisms in genetic association studies.

The measurement methods also demonstrated diversity. Immunohistochemistry (IHC) was the primary approach employed to localize and semiquantify eNOS expression in endometrial tissues, supported by many studies integrating IHC with real-time PCR to validate transcriptional changes. Genotyping techniques, including PCR-RFLP and sequencing, were employed to identify prevalent NOS3 variations, particularly Glu298Asp and intron 4 VNTR 4a/4b, while a biochemical assessment of nitric oxide metabolites was conducted using colorimetric or Griess-based assays.

The measured results were categorized into two primary groups: molecular outcomes, such as eNOS protein expression, NO metabolite levels, and transcriptional responses of target genes, and clinical reproductive outcomes, including implantation rates, biochemical or clinical pregnancy, miscarriage, and IVF success or failure. Some studies focused solely on endometrial molecular markers during the implantation window, whereas others specifically linked nitric oxide dysregulation to adverse reproductive outcomes. Despite variations in demographics and methodology, a constant finding across multiple studies was the dysregulated expression of eNOS in the endometrium of women facing reproductive failure. Najafi et al. (2012, 2013) and Banerjee et al. (2013) demonstrated significantly elevated eNOS expression in the luminal and glandular epithelial cells of women with urinary incontinence and recurrent pregnancy loss, respectively. These findings support the hypothesis that excessive nitric oxide production may lead to nitrosative stress, cellular apoptosis, or inadequate blastocyst adhesion [16,20,22]. Conversely, highlighting potential ethnic or methodological variety, Makino et al. (2004) and Karvela et al. (2008) failed to establish statistically significant relationships between NOS3 polymorphisms and RPL [23,25].

In contrast, studies assessing NO_2_^−^/NO_3_^−^ concentrations in serum or follicular fluid yielded inconsistent results. Roychoudhury et al. (2016) demonstrated elevated serum eNOS in women with repeated implantation success relative to those with failure [33]; however, Fábregues et al. (2000) observed no correlation between systemic nitrite/nitrate levels and implantation success [26]. Such discrepancies may be attributed to differences in the analytical sensitivity of the employed assays, patient selection criteria, or the time of sample collection. The experimental modification of the NO pathway was also investigated. Melford et al. (2021) demonstrated that nitric oxide donors altered endocannabinoid-related gene expression in receptive endometrial cells, suggesting a potential molecular mechanism for nitric oxide-enhanced receptivity [15]. Conversely, Ohl et al. (2002) evaluated a clinical intervention employing nitroglycerin patches during embryo transfer but found no statistically significant enhancement in pregnancy rates [36].

Table 4 illustrates the methodological diversity and mechanistic intricacy present in the included studies. It provides a systematic overview of the role of nitric oxide signaling—via eNOS activity, NO metabolite concentrations, or genetic polymorphisms—in both normal and pathological implantation processes. This foundation organizes the subsequent theme synthesis into mechanistic subdivisions.

### 3.2. The Expression of Endothelial Nitric Oxide Synthase in the Endometrium

The aberrant expression of eNOS in the endometrium of women experiencing implantation failure, persistent infertility, and recurrent miscarriage is a consistent and mechanistically significant finding across the studies reviewed. The window of implantation typically occurs between days 20 and 24 of a standard menstrual cycle, characterized by a complex interplay of molecular and hormonal signals that render the endometrium receptive. NO, mostly produced by eNOS, is crucial in this microenvironment as a multifaceted signaling molecule that orchestrates tissue remodeling, immune control, and vascular flexibility.

eNOS is continuously expressed in the luminal and glandular epithelium, as well as in the uterine vascular endothelium; its activity is meticulously regulated by sex steroid hormones and intracellular calcium fluctuations. The activation of eNOS by progesterone induces the localized release of NO, which functions through the sGC–cGMP pathway to facilitate vasodilation and improve endometrial blood flow during the mid-secretory phase. NO is essential for extracellular matrix remodeling and the transformation of spiral arteries since it facilitates the upregulation of MMPs and VEGF. Furthermore, nitric oxide regulates immunological tolerance by modulating the activity of macrophages and uterine natural killer (uNK) cells, thereby facilitating mother–fetus contact.

This framework indicates that both insufficient and excessive production of NO may adversely affect endometrial receptivity. Najafi et al. (2012, 2013) and Banerjee et al. (2013) demonstrated significantly elevated eNOS expression in endometrial biopsies obtained during the implantation window from women with UI and recurrent RPL, respectively. RT-qPCR analysis and immunohistochemistry labeling confirmed that the luminal epithelial layer, where initial blastocyst apposition occurs, exhibits the highest level of upregulation. The data suggest that elevated nitric oxide production at the epithelial surface may disrupt the expression of adhesion molecules such as integrins αvβ3 and L-selectin ligands, hence impairing embryo–endometrium communication and reducing implantation potential [16,20,22].

In addition to adhesion, NO has been shown to induce nitrosative stress mostly through ONOO^−^, a reactive nitrogen species generated by the interaction of NO with O_2_^−^. Peroxynitrite can induce lipid peroxidation, DNA damage, and mitochondrial dysfunction, leading to the death of endometrial epithelial and stromal cells, thus jeopardizing the viability of the pre-implantation embryo. Banerjee et al. observed microvascular changes in the endometrium of women with recurrent pregnancy loss, including congested and dilated capillaries, suggesting that sustained excess nitric oxide may undermine endothelial integrity and decidual angiogenesis, thereby disrupting the peri-implantation environment.

In contrast, Makino et al. (2004) observed no significant difference in eNOS expression between fertile controls and women experiencing pregnancy loss. They found elevated systemic levels of NO_2_^−^/NO_3_^−^ in women who experienced miscarriage, indicating that eNOS-derived NO may be functionally uncoupled due to oxidative stress or that systemic NO metabolism may be enhanced independently of local endometrial eNOS expression. This outcome aligns with recent studies indicating that post-translational changes, tetrahydrobiopterin (BH_4_) availability, and oxidative balance influence the enzyme’s coupling status and the generation of nitric oxide vs. superoxide, hence affecting eNOS activity relative to its expression level [25].

Sun et al. (2003) performed experimental studies specifically examining the hormonal regulation of eNOS. In this framework, luteal phase mifepristone—a progesterone receptor antagonist—was administered to fertile women. This intervention significantly diminished eNOS expression in glandular epithelial cells, thereby substantiating the hypothesis that progesterone enhances eNOS during the receptive phase and that the disruption of this hormonal signal constrains eNOS-mediated endometrial metamorphosis. ENOS regulates prostaglandin synthesis, VEGF signaling, and stromal decidualization; thus, its inhibition by progesterone blockade may significantly impact implantation competence [35].

The study by Wang et al. (2006), focusing on women with infertility related to endometriosis, provides more molecular insights. These patients had persistently elevated eNOS expression throughout the menstrual cycle, likely due to oxidative stress, inflammation characteristic of endometriosis, and extended estrogenic stimulation. Subsequent to GnRH agonist therapy, which reduced peripheral estrogen levels and suppressed pituitary gonadotropins, eNOS expression returned to normal levels, and an enhancement in endometrial histology was seen. These findings substantiate the notion that hyperestrogenic circumstances may lead to pathological overactivity of eNOS, hence fostering an inhospitable endometrial environment [34].

At the molecular level, eNOS-derived nitric oxide modulates transcription factors such as NF-κB, HIF-1α, and PPAR-γ through cGMP and S-nitrosylation of target proteins. Dysregulated S-nitrosylation can lead to atypical gene expression associated with apoptosis, immune response, and matrix remodeling. Additionally, eNOS engages with intracellular scaffolding proteins that modulate its enzymatic activity, including heat shock protein 90 (Hsp90) and caveolin-1. The disruption of these interactions in pathological conditions may elucidate anomalous NO signaling and inadequate implantation.

These tests collectively demonstrate the critical significance of precise spatial and temporal regulation of eNOS activity in maintaining a responsive endometrium. Overexpression or dysregulation of eNOS disrupts many signaling pathways associated with immunological homeostasis, cellular survival, vascular remodeling, and embryo–maternal contact. Consequently, anomalous nitric oxide production may serve as a prevalent downstream pathway linking many infertility phenotypes—such as recurrent pregnancy loss, unexplained infertility, and endometriosis—with suboptimal implantation results.

### 3.3. NO2^−^/NO_3_^−^ Concentrations and IVF Outcomes (Elaborated—Molecular Perspective)

NO serves as a transient signaling molecule having a half-life of only a few seconds. NO swiftly converts into NO_2_^−^ and NO_3_^−^ by a series of redox reactions, owing to its quick reactivity with oxygen and superoxide. These enduring oxidative derivatives serve as repositories of bioactive nitric oxide and as indicators for endogenous nitric oxide production, particularly in hypoxic or acidic conditions where nitrate–nitrite–nitric oxide reduction pathways are reactivated through both enzymatic and non-enzymatic mechanisms.

Nitrite and nitrate production at the molecular level transpires directly downstream of eNOS enzymatic activity, necessitating L-arginine, oxygen, and several cofactors, including BH_4_. ENOS produces nitric oxide in a tightly regulated manner during homeostasis. ENOS may become uncoupled in pathological conditions, such as oxidative stress, inflammation, or hormonal dysregulation, resulting in decreased NO and increased O_2_^−^ production. This promotes the formation of ONOO^−^, hence causing oxidative damage to nucleic acids and lipids, enzyme inactivation, and the nitration of tyrosine residues.

In this regard, the measurement of NO_2_^−^ and NO_3_^−^ can indirectly provide a functional representation of NO pathway activity. Numerous studies have been undertaken to correlate the systemic levels of these metabolites with implantation outcomes in women undergoing IVF. Roychoudhury et al. (2016) demonstrated that pregnant women exhibited serum NO_2_^−^/NO_3_^−^ levels exceeding those of non-pregnant controls. This discovery corroborates the hypothesis that successful implantation necessitates an optimal level of nitric oxide bioavailability—neither insufficient nor excessive. It signifies the vasodilatory, angiogenic, and immunoregulatory functions of nitric oxide inside the peri-implantation endometrium [33].

The activation of sGC and the subsequent increase in cGMP facilitate NO-induced vasodilation. cGMP activates PKG, resulting in increased endometrial perfusion and the modulation of endothelial barrier function, hence inducing the relaxation of vascular smooth muscle. Furthermore, NO regulates the expression of angiogenic factors, including VEGF-A, and modulates matrix metalloproteinases (MMP-2 and MMP-9), which are critical for extracellular matrix remodeling and trophoblast invasion.

In addition to its vascular actions, nitric oxide also influences inflammatory tone and immunological tolerance. NO enhances the recruitment and preservation of regulatory T cells (Tregs) and tolerogenic dendritic cells, essential for establishing immunological quiescence at the maternal–fetal interface, while simultaneously diminishing pro-inflammatory cytokines such as TNF-α and IL-1β in healthy conditions. Consequently, the concentrations of NO_2_^−^ and NO_3_^−^ may serve as systemic indicators of endometrial immunological readiness.

Conversely, Fábregues et al. (2000) found no link between IVF success and levels of NO metabolites. This discrepancy may be attributed to the inability of systematic NO_2_^−^/NO_3_^−^ measurements to identify localized NO signaling within the uterine environment. The endometrium has segmented nitric oxide production, as stromal, epithelial, and endothelial compartments respond differentially to hormonal inputs. The half-life and bioavailability of nitric oxide metabolites are additionally affected by renal clearance, plasma antioxidant capacity, and dietary nitrate intake, complicating the interpretation of peripheral data [26].

Although eNOS tissue expression remained unchanged, research by Makino et al. (2004) identified elevated NO_2_^−^/NO_3_^−^ levels in women undergoing early pregnancy loss. In inflammatory disorders, this paradox frequently indicates a transition from eNOS-dependent nitric oxide production to iNOS-mediated nitric oxide production. In contrast to eNOS, iNOS is transcriptionally upregulated in response to NF-κB activation, LPS, or IFN-γ, resulting in either lethal effects or elevated, sustained amounts of NO. Excessive iNOS activity and the resultant accumulation of NO metabolites signify a dysregulated inflammatory condition that undermines implantation by causing decidual cell loss, vascular instability, and oxidative damage [25].

From a systems biology perspective, nitric oxide and its metabolites are essential components of the redox regulatory network that equilibrates pro-survival and pro-apoptotic signals. This equilibrium involves interaction with the Nrf2 pathway, which induces the production of HO-1 and SOD as antioxidant response components. Nrf2 activation may be compromised under nitrosative stress, resulting in oxidative damage and impaired tissue remodeling essential for implantation. Moreover, during hypoxia, a critical phase in early implantation, nitric oxide metabolites influence HIF-1α stability. Hypoxia-induced HIF-1α promotes stromal cell proliferation, glycolysis, and angiogenesis, all of which are concurrently enhanced by NO signaling. Fluctuations in NO_2_^−^/NO_3_^−^ concentrations induce disturbances in this interaction, perhaps resulting in a disorganized cellular response to hypoxia, and thus constraining receptivity.

Considering these findings, NO_2_^−^ and NO_3_^−^ may possess properties beyond being mere inert end-products. Recent investigations indicate that xanthine oxidase, aldehyde oxidase, and maybe mitochondrial respiratory enzymes serve as enzymatic routes that, under acidic and hypoxic circumstances characteristic of the peri-implantation endometrium, convert these anions back to nitric oxide. In impaired uteri, this nitrate–nitrite–NO pathway provides an alternative, oxygen-independent mechanism for NO synthesis that may compensate for diminished eNOS activity. From a molecular pathology standpoint, NO_2_^−^ and NO_3_^−^ serve as significant contributors to redox biology, immunological regulation, and vascular remodeling in the reproductive tract rather than merely functioning as metabolic indicators. To accurately assess endometrial receptivity, it is essential to evaluate their levels in conjunction with markers of eNOS/iNOS equilibrium, oxidative stress, and hormonal control.

### 3.4. NOS3 Gene Polymorphisms and Reproductive Failure

The NOS3 gene, located on chromosome 7q36.1, encodes endothelial eNOS, an essential enzyme responsible for the continuous production of NO in vascular and reproductive tissues. All of these factors are essential for successful embryo implantation; eNOS-derived NO regulates vascular tone, angiogenesis, immune function, and cellular signaling. Polymorphisms in the NOS3 gene can alter eNOS synthesis, enzymatic activity, mRNA stability, or protein structure, hence influencing NO bioavailability and subsequent signaling in the endometrium. Such alterations could disrupt the meticulously calibrated NO signaling network essential for establishing a receptive endometrial environment. This review aims to elucidate the impact of genetic vulnerability on endometrial receptivity through nitric oxide dysregulation, examining the correlation between NOS3 genetic variants and implantation failure, recurrent RPL, and unexplained infertility. Understanding these interactions may identify novel targets for therapeutic intervention in assisted reproduction and biomarkers for diagnosis.

The often-studied variants include the Glu298Asp polymorphism (rs179998) in exon 7, resulting from a G to T substitution (894G>T), and the variable number of tandem repeats (VNTR) in intron 4, commonly referred to as the 4a and 4b alleles. At position 298, the Glu298Asp variant replaces glutamic acid with aspartic acid, thereby undermining the structural integrity of eNOS, enhancing proteolytic cleavage, and diminishing enzymatic stability. This structural alteration may reduce NO synthesis under physiological stimuli and hinder the ability of eNOS to form dimers, which are essential for enzymatic function. Tissues reliant on precisely regulated nitric oxide signaling, such as the endometrium, may experience compromised vasodilation, reduced perfusion, and the disruption of immunological homeostasis, hence impacting implantation success.

The Glu298Asp variant is associated with diminished vascular responsiveness, endothelial dysfunction, and increased susceptibility to cardiovascular disease. Hefler et al. (2002) discovered that women homozygous for the Asp298 gene exhibited a significantly higher incidence of recurrent miscarriage, suggesting that diminished NO signaling may adversely affect endometrial perfusion, decidualization, or early placental development. The two characteristics of a responsive endometrium, namely spiral artery remodeling and stromal cell transformation, may be inhibited by diminished nitric oxide levels. Women with unexplained infertility exhibit analogous relationships, indicating that diminished nitric oxide availability may hinder the vascular and immunological modifications essential for successful implantation. The disruption of these pathways may hinder appropriate embryo–endometrium communication, potentially resulting in either early developmental arrest or implantation failure [24].

The VNTR polymorphism in intron 4, consisting of either four (4a) or five (4b) tandem 27 bp repeats, may affect NOS3 pre-mRNA splicing and transcriptional efficiency. Research indicates that reduced eNOS expression and diminished NO production are associated with the 4a allele. Decreased eNOS mRNA levels may arise from alterations in transcription factor binding affinity associated with intronic polymorphism or from modifications in enhancer activity. This genetic variant may predispose individuals to implantation failure by limiting the vasodilatory and immunomodulatory functions of nitric oxide, resulting in endometrial hypoperfusion, inadequate stromal remodeling, and insufficient leukocyte recruitment. Furthermore, this may diminish uterine receptivity by altering the ratio of inflammatory to anti-inflammatory cytokines, which is crucial for embryo tolerance.

NOS3 polymorphisms may influence critical signaling pathways at the molecular level. Decreased NO production induced by Glu298Asp or 4a variants alters sGC/cGMP/PKG signaling, thereby downregulating genes associated with angiogenesis (e.g., VEGF-A), cell adhesion (e.g., integrin αvβ3), and matrix remodeling (e.g., MMP-9). The cGMP-dependent PKG pathway is crucial for smooth muscle relaxation, endothelial cell proliferation, and the transcription of anti-inflammatory genes. Moreover, diminished NO availability may lead to insufficient control of pro-inflammatory transcription factors, such as NF-κB, thus establishing a detrimental immunological environment for the embryo. The inflammatory bias contributes to implantation failure by inducing cytotoxic responses, increasing oxidative stress, and impairing trophoblast penetration.

At the epigenetic level, NOS3 polymorphisms may interact with the methylation patterns of the NOS3 promoter, hence influencing gene expression in a tissue-specific manner. Transcription factor accessibility or SNP-dependent chromatin remodeling may alter the DNA methylation of CpG islands in the promoter region. Furthermore, individuals with NOS3 variants may alter the post-translational regulation of eNOS, including phosphorylation at Ser1177, acylation, and protein–protein interactions with caveolin-1 and Hsp90, thereby intensifying the effects on NO signaling. These modifications affect enzyme sensitivity to shear stress and hormonal cues, as well as stability and location. Genetic differences may influence not just NOS3 transcription but also the intricate network of post-transcriptional and post-translational regulation.

Environmental and hormonal factors appear to regulate the penetrance of these polymorphisms. Estrogen, a known enhancer of NOS3 transcription through ERα activation, may assist individuals with hypomorphic alleles in partially compensating for reduced enzymatic activity. In scenarios of estrogen dominance (e.g., endometriosis), this compensatory strategy may become maladaptive, resulting in eNOS overexpression, oxidative stress, and endometrial dysfunction. Through PR-mediated signaling, progesterone may influence eNOS activity and nitric oxide equilibrium. Therefore, while evaluating the clinical significance of NOS3 polymorphisms, it is essential to consider gene–environment interactions, encompassing endocrine, nutritional, and immunological influences.

The therapeutic significance of NOS3 polymorphisms remains under extensive investigation. While several studies, including those by Karvela et al. (2008) and Makino et al. (2004), have failed to demonstrate statistically significant connections between NOS3 genotypes and pregnancy loss, others revealed notable associations between certain alleles and diminished reproductive success. Ethnic diversity, a limited sample sizes, or insufficient functional validation may all contribute to these discrepancies. Additionally, variances in outcomes may stem from differences in outcome definitions, inclusion criteria, and laboratory methodologies among studies. Determining the extent to which NOS3 polymorphisms independently forecast implantation failure or interact with other molecular risk factors will require meta-analyses and mechanistic investigations [23,25].

In people with recurrent implantation failure or idiopathic infertility, genetic screening for NOS3 mutations may offer predictive insights within the context of IVF. Moreover, pharmacological interventions aimed at reinstating NO signaling—such as L-arginine supplementation, BH_4_ cofactor therapy, or NO donors—could be tailored according to individual NOS3 genotypes, thereby facilitating personalized strategies to enhance endometrial receptivity. Future treatments to optimize endometrial function in genetically predisposed patients may involve targeted gene editing, epigenetic alteration, or the administration of eNOS-regulating microRNAs. Incorporating NOS3 genotyping into reproductive diagnostics holds significant potential for risk stratification and personalized treatment.

### 3.5. Modulation of Experimental Pathways 

Significant mechanistic insights into the influence of NO on endometrial receptivity and implantation are shown in experimental studies that manipulate the NO pathway. The effects of in vitro or in vivo interventions on significant reproductive outcomes have been examined through the augmentation or inhibition of nitric oxide production. These studies enhance our understanding of nitric oxide as a dynamically controlled signal with potential therapeutic applications in infertility rather than only as a passive mediator. The direct alteration of the components of the NO signaling axis has elucidated their role in orchestrating hormonal responses, immunological interactions, and tissue remodeling within the peri-implantation endometrium. This experimental modification serves as a platform for evaluating potential therapeutic medications or delivery systems designed to rectify nitric oxide dysregulation in pathological conditions such as endometrial insufficiency or recurrent implantation failure.

Ohl et al. (2002) conducted a significant study on nitric oxide modulation, evaluating the effects of transdermal nitroglycerin patches—a known nitric oxide donor—on women undergoing in vitro fertilization embryo transfer. The vasodilatory effects of nitric oxide were cited as the rationale as they may enhance uterine perfusion and facilitate implantation. The study raised important inquiries on the timing, dosage, and individual responsiveness to exogenous NO, although not demonstrating a statistically significant increase in clinical pregnancy rates. The absence of a significant effect may have stemmed from nitroglycerin’s systemic rather than localized action, perhaps diminishing its impact on the endometrial vasculature. It emphasized the challenge of transforming systemic nitric oxide donors into localized, tissue-specific effects within the endometrium. The potential for NO overexposure and ensuing nitrosative stress must be meticulously weighed against their physiological benefits [36]. Future studies should examine whether minimizing systemic deleterious effects and enhancing localized endometrial receptivity through targeted intrauterine nitric oxide release or receptor-specific nitric oxide modulation could yield superior outcomes.

In vitro models have facilitated more precise mechanical comprehension. Melford et al. (2021) examined the impact of pharmacological nitric oxide donors on gene expression associated with receptivity in cultured human endometrial epithelial cells. Indicating that NO engages with lipid signaling networks critical for embryo–endometrium communication, they demonstrated that NO exposure modulated genes involved in endocannabinoid metabolism, such as fatty acid amide hydrolase (FAAH) and N-acyl phosphatidylethanolamine-specific phospholipase D (NAPE-PLD). Notably, NO appeared to diminish pro-inflammatory mediators while enhancing markers associated with a responsive phenotype, such as HOXA10 and LIF. These findings underscore the significance of nitric oxide as a modulator of endometrial gene networks and reveal its potential influence on the molecular environment of the receptive endometrium. The relationship with the endocannabinoid system is particularly compelling because of its emphasis on nitric oxide’s role in synchronizing neuroimmune signaling pathways that influence implantation. Moreover, the implication of NO as an epigenetic modulator is evidenced by its influence on chromatin remodeling and the transcriptional activation of genes associated with implantation, thereby further integrating endocrine and environmental signals in the establishment of endometrial receptivity [15].

From a signaling standpoint, the sGC/cGMP/PKG axis is a traditional downstream target demonstrated by experimental modification of the NO pathway. The activation of this cascade enhances vascular permeability, stromal cell decidualization, and cytoskeletal reorganization—characteristics of a functionally sensitive endometrium. The cGMP-dependent PKG reinforces the structural and functional adaptations of the endometrium by phosphorylating several target proteins involved in cellular adhesion, migration, and vascular integrity. Additionally, there is an interaction between NO signaling and ER pathways in which NO enhances ERα activity and promotes the expression of progesterone-induced decidual genes. This cumulative influence indicates that throughout the implantation window, nitric oxide works as a hormone amplifier. The endometrium is prepared for optimal embryo implantation through the integration of estrogen receptor and nitric oxide-dependent transcriptional networks, highlighting the importance of hormonal and redox equilibrium for reproductive success.

Moreover, providing insights into its essential roles involves the experimental inhibition of NO generation. In endometrial cultures, the pharmacological suppression of eNOS by L-NAME results in diminished angiogenesis, heightened epithelial apoptosis, and modified stromal–epithelial communication. These findings illustrate the histological patterns observed in infertile endometria and substantiate the hypothesis that the development of endometrial receptivity is contingent upon endogenous NO production. In addition to angiogenic deficits, nitric oxide suppression influences the secretion of vital cytokines and growth factors, such as VEGF, IL-15, and IGFBP-1, all of which are essential for sustaining stromal decidualization and embryo–maternal communication. The loss of NO further exacerbates cellular senescence and impairs tissue remodeling, resulting in increased oxidative stress and mitochondrial dysfunction. These findings validate the dual role of nitric oxide as both a structural and signaling mediator in the endometrium, hence orchestrating the hormonal, immunological, and vascular signals essential for implantation.

Comprehensive experimental research indicates that the modulation of nitric oxide—through either donor administration or enzymatic inhibition—affects critical molecular and cellular processes essential for implantation. Despite the limited therapeutic efficacy of systemic nitric oxide donors due to their non-specificity and dosing challenges, localized or targeted approaches such as intrauterine nitric oxide-releasing gels or nanocarriers may exhibit promising possibilities in the future. Furthermore, integrating NO-based therapies with hormonal or antioxidant co-treatments may optimize outcomes in women experiencing implantation failure due to oxidative or endothelial dysfunction. To enhance therapeutic precision, individualized nitric oxide modulation strategies could potentially incorporate endometrial gene expression patterns and genetic background, such as NOS3 polymorphisms. Particularly in IVF cohorts experiencing recurrent implantation failure or diminished endometrial thickness, these integrated strategies may enhance the precision and effectiveness of nitric oxide-based therapies. The advancement of biomaterial-based delivery devices that mimic physiological nitric oxide gradients and release kinetics offers a promising future in reproductive health.

## 4. Discussion

This review systematically summarizes evidence from 14 studies evaluating the role of NO, its metabolites NO_2_^−^/NO_3_^−^, and eNOS in endometrial receptivity and implantation outcomes. It is composed of publications that include mechanistic in vitro investigations, randomized clinical trials, case–control and cohort designs, and other methodologies. The findings collectively underscore the essential role of the NO pathway in orchestrating the molecular and cellular processes fundamental to successful implantation.

The aberrant expression of eNOS in the endometria of women with RPL, UI, and RIF was a consistent finding. Numerous studies indicated that eNOS was overexpressed in the luminal epithelium during the implantation window, suggesting that excessive NO production could lead to diminished blastocyst adhesion or heightened nitrosative stress. Conversely, several studies emphasized the potential consequences of reduced nitric oxide bioavailability, particularly in women possessing NOS3 gene polymorphisms such as Glu298Asp and intron 4 VNTRs, which are associated with diminished endothelial nitric oxide synthase activity and impaired endometrial vascular function.

The findings regarding serum or plasma NO_2_^−^ and NO_3_^−^ as systemic markers were inconsistent. Certain studies revealed elevated levels in women who underwent successful implantation or experienced pregnancy loss, suggesting that systemic nitric oxide metabolites may either indicate or alter endometrial nitric oxide signaling. Nonetheless, the lack of correlation in additional studies highlights the constraints of depending on peripheral NO_2_^−^/NO_3_^−^ as reliable indicators of local endometrial activity.

The experimental modification of NO signals has ultimately confirmed its functional significance. The reduction in nitric oxide synthesis hindered stromal decidualization and promoted cell death, whereas the in vitro supplementation of nitric oxide enhanced the expression of receptivity markers and angiogenic genes. The challenges of translating biological discoveries into therapeutic advantages, in the absence of targeted delivery systems, are evidenced by the absence of enhancement in IVF outcomes from a solitary randomized trial of NO donor treatment (nitroglycerin).

The data indicate that both insufficient and excessive NO signaling disrupt endometrial homeostasis, highlighting the necessity for precise temporal and spatial regulation of eNOS-derived NO for optimal receipt. The findings also suggest potential targets for personalized therapeutic interventions in reproductive medicine and a biomarker for implantation failure due to NO pathway dysregulation.

### 4.1. Interpretation of Findings and Molecular Mechanisms

The research concurs on the significant role of NO as a multifaceted regulator of endometrial receptivity; thus, both excessive and insufficient NO signaling can disrupt the intricate molecular and cellular processes essential for successful implantation [15]. Najafi et al. demonstrated that elevated local nitric oxide production could impair embryo adhesion through nitrosative stress and the downregulation of adhesion molecules like integrins, as evidenced by the overexpression of eNOS in the luminal and glandular epithelial cells of women experiencing unresolved infertility and recurrent miscarriage [16]. Using InvIRM, Banerjee et al. observed increased eNOS immunoreactivity alongside altered vascular architecture, indicating that abnormal NO-mediated vascular remodeling may be detrimental in early pregnancy loss [22]. These findings underscore the pro-oxidant capacity of nitric oxide at supraphysiological levels, aligning with Su et al.’s demonstration that excessive nitric oxide induces mitochondrial dysfunction, ROS production, and the activation of apoptotic pathways in reproductive tissues [35].

Agarwal et al. elucidated the adverse effects of nitrosative stress on the reproductive tract, encompassing lipid peroxidation, diminished cytokine signaling, and immunological dysregulation inside the endometrium, thus reinforcing this notion. These alterations are thought to disrupt the immune–epithelial interaction essential for embryo tolerance and implantation [18]. Budani et al. expanded this framework by proposing that nitric oxide serves as a mediator between oxidative stress and endothelial dysfunction, and when dysregulated, may disrupt the angiogenic and decidualizing systems essential for implantation competence [38]. Al Sallout et al. and Loizidou et al. underscored its vasodilatory and protective functions in physiological conditions while also noting its potential to induce endothelial injury, generate pro-inflammatory cytokines, and promote aberrant neovascularization under chronic oxidative stress, thereby affirming the dualistic nature of nitric oxide (NO) [31,32].

Zullino et al. proposed that NO establishes an epigenetically active signaling network in the endometrial milieu through its interaction with EVs and exosomal microRNAs, hence exerting regulatory impact. The authors suggested that NO can regulate the payload of EVs, encompassing members of the miR-21 and miR-200 families, thereby modifying gene expression in target cells, including stromal and epithelial cells [39]. Through coordinated effects on apoptosis, local immunological tolerance (such as Treg and NK cell modulation), and redox-sensitive transcription factors like NF-κB and HIF-1α, Andronico et al. and Muraoka et al. suggested that NO may regulate trophoblast–endometrium communication [40,41].

Collectively, these findings present a cohesive framework in which nitric oxide serves as a central molecular integrator, linking endometrial receptivity to vascular tone, immune modulation, redox balance, and gene expression. Nitric oxide, under precise regulation, facilitates critical processes in implantation such as leukocyte recruitment, decidualization, angiogenesis, and trophoblast invasion [42]. Dysregulated eNOS, whether through overexpression, uncoupling due to oxidative stress, or inadequate NO synthesis from environmental or genetic factors, acts as a disruptor of implantation, leading to compromised perfusion and potential immunological rejection of the embryo [43]. Histological, molecular, and in vitro evidence demonstrates that nitric oxide homeostasis is a crucial determinant of reproductive success.

In contrast to studies emphasizing local endometrial eNOS overexpression, other research highlighted the significance of systemic NO metabolite levels, suggesting either an alternative or complementary mechanism of dysregulation [44]. Makino et al. observed no significant variation in endometrial eNOS protein expression between fertile controls and women undergoing early pregnancy loss; nonetheless, they recorded markedly elevated blood levels of NO_2_^−^. This outcome suggests that excessive systemic nitric oxide synthesis, potentially through iNOS activity in circulating immune or endothelial cells, may lead to implantation failure due to nitrosative stress, systemic inflammation, or vascular dysfunction beyond the uterine environment. Comparable assessments of serum NO_2_^−^/NO_3_^−^ in women undergoing IVF demonstrated no association with implantation success, suggesting that peripheral NO metabolites may not accurately represent localized NO activity inside the endometrium. These findings underscore the compartmentalization of nitric oxide signaling, suggesting that systemic assessments may fail to identify temporal and geographical variations at the embryo–endometrial interface [25].

However, not all findings support the inconsistency between local and systemic nitric oxide activity. Roychoudhury et al. discovered that women who achieved successful implantation and clinical pregnancy after IVF exhibited significantly elevated serum NO_2_^−^/NO_3_^−^ levels compared to non-pregnant women, suggesting that systemic NO availability may indicate a suitable vasodilatory and redox environment conducive to a receptive endometrium. This duality underscores the necessity for a contextual interpretation of nitric oxide metabolite data: elevated nitric oxide levels may indicate robust perfusion and immunological tolerance, or conversely, reflect pro-inflammatory inducible nitric oxide synthase activity in subclinical disease states [33].

The hormonal regulation of eNOS introduces an additional layer of complexity. Sun et al. demonstrated that mifepristone, a progesterone receptor antagonist, significantly decreased eNOS expression in endometrial glandular epithelial cells, therefore emphasizing the dependence of eNOS activation on luteal-phase progesterone signaling [35]. Additional evidence is provided by the research conducted by Chwalisz et al., which showed that nitric oxide (NO) exerts a significant downstream effect on steroid hormone receptor function during the implantation period. Essential for successful blastocyst invasion, they elucidated the involvement of nitric oxide in the decidualization process, stromal–epithelial interactions, and vascular remodeling [45]. Battaglia et al. elaborated on these concepts by linking dynamic fluctuations in eNOS expression and NO bioavailability throughout the menstrual cycle to cyclical hormonal alterations, particularly the estrogen–progesterone equilibrium. They suggested that the hormonal environment regulates both nitric oxide generation and the endometrium’s response to oxidative stress and immunological activation [46].

These data collectively underscore the interplay between the hormonal regulation of eNOS expression, systemic rather than localized control of NO, and the inflammatory milieu surrounding implantation [47]. While elevated systemic NO levels may indicate a beneficial vasoreactive milieu, their interpretation must consider redox status, eNOS versus iNOS, and hormonal synchronization [48]. The necessity for spatiotemporal precision in assessing NO-related biomarkers in clinical settings determines whether NO acts as a facilitator or inhibitor of implantation based on the functional balance between these factors.

The data indicate that NO serves as a crucial upstream regulator of many signaling cascades pertinent to implantation. NO activates sGC, resulting in the synthesis of cGMP and the activation of PKG [49]. This pathway regulates cytoskeletal rearrangement, vascular tone, and endothelial integrity. Budani et al. emphasized that the sGC–cGMP axis facilitates angiogenic signaling through the phosphorylation of endothelial targets that regulate cell proliferation and permeability—essential activities for endometrial vascular remodeling during the receptive phase. Furthermore, NO–cGMP signaling promotes the decidual transformation of stromal cells, a process essential for successful implantation [38].

NO further downstream regulates the expression of angiogenic factors, including VEGF-A, which promotes capillary remodeling and neovascularization inside the endometrial functional layer [50]. Wang et al. demonstrated that eNOS overexpression coupled with aberrant vascular morphology corresponded with VEGF dysregulation, which was rectified after GnRH agonist therapy in individuals with endometriosis-related infertility. This substantiates the hypothesis that NO influences VEGF expression either directly through transcriptional pathways or indirectly by modulating hypoxia signaling. The absence of availability also affects HIF-1α, the principal regulator of cellular response to hypoxia. Insufficient NO may induce S-nitrosylation of HIF-related coactivators, hence obstructing angiogenesis, whereas physiological NO levels sustain HIF-1α and enhance VEGF production [34].

NO concurrently influences matrix remodeling via regulating matrix metalloproteinMMP-2 and MMP-9, enzymes responsible for degrading extracellular matrix constituents and facilitating trophoblast invasion [51]. Altered nitric oxide signaling, as noted by Su et al. and Agarwal et al., disrupts matrix metalloproteinase expression patterns, potentially leading to superficial implantation and an increased risk of early loss. Moreover, integrin αvβ3, an essential adhesion protein, is regulated by NO levels and is expressed on the uterine luminal epithelium during the implantation window. Low NO may reduce integrin gene transcription, both of which are associated with inadequate apposition and adhesion of the blastocyst; conversely, excessive NO has been shown to impair integrin clustering and functionality [18,52].

Additionally, NO engages with immune-regulatory pathways to modulate the expression of cytokines such as IL-10, TGF-β, and IL-15, thereby orchestrating the functions of macrophages and uterine natural killer (uNK) cells [53].

Muraoka et al. and Andronico et al. emphasized the immunological role of nitric oxide, highlighting its influence on immune tolerance at the mother–fetus interface, partly through its impact on the epigenetic regulation of immune-related genes via microRNA networks. The immunological effects are vital as early placentation and implantation may be hindered by even slight fluctuations in pro- versus anti-inflammatory cytokines [40,41].

### 4.2. Functional Implications and Genetic Regulation: NOS3 Polymorphisms

Early pregnancy loss, RIF, and unexplained infertility predominantly stem from a genetic predisposition to NO dysregulation [54]. The most well-studied genetic variations in the NOS3 gene, which encodes eNOS, include the 27 bp variable number tandem repeat (VNTR) polymorphism in intron 4 and the Glu298Asp single nucleotide polymorphism (rs179998) [55].

These genetic variations are believed to undermine eNOS mRNA stability, protein conformation, or transcriptional regulation, hence influencing enzyme activity and nitric oxide bioavailability [56]. The Glu298Asp polymorphism results in an amino acid substitution from glutamate to aspartate at position 298, hence increasing proteolytic sensitivity and reducing dimer formation, which is essential for enzymatic activity [57]. The VNTR polymorphism can impact gene expression through modifications in chromatin conformation or variations in alternative splicing efficiency [58]. All of these factors are essential for implantation; these changes have been collectively associated with low vascular tone, diminished endometrial perfusion, and insufficient stromal decidualization.

The initial clinical data originates from the study conducted by Hefler et al., which identified that women experiencing recurrent miscarriage are more predisposed to possess the Asp298 allele. Their findings indicate that minor alterations in eNOS activity could influence the uterine milieu during early gestation [24]. Karvela et al. and Makino et al. examined analogous polymorphisms in women with unexplained infertility, observing altered genotype distributions, likely attributable to variations in sample size or ethnicity [23]. Budani et al. provided a comprehensive mechanistic review of the sGC–cGMP–PKG pathway, which regulates endothelial proliferation, vascular tone, and inflammatory homeostasis, demonstrating how these variants modify downstream NO-dependent signaling. It is suggested that disturbance in this cascade affects endometrial receptivity and the remodeling of spiral arteries. Furthermore, these differences may alter the regulation of MMPs, thus undermining the endometrium’s ability to remodel and facilitate embryo invasion [38].

Recent translational studies affirm the pathophysiological significance of nitric oxide dysregulation. Voros et al. (2024) demonstrated that bariatric surgery in obese women reactivates the nitrate–nitrite–NO pathway and diminishes oxidative stress, a common consequence of diminished NOS3 function, therefore restoring NO signaling and enhancing reproductive results. Their systematic investigation emphasized the necessity of reestablishing metabolic and vascular equilibrium, as well as the capacity of targeted environmental or surgical interventions to partially mitigate genetic predisposition. This emphasizes the concept of gene–environment interactions in reproductive health, wherein NO-related genotypes may regulate individual responses to external stimuli [27].

Additional mechanistic data is provided by Banerjee et al. (2013), who showed that in women with idiopathic recurrent miscarriage, diminished endometrial expression of eNOS, VEGF, and IL-10 was associated with inadequate subendometrial blood flow. Their application of cytokine quantification and Doppler ultrasound imaging directly linked cellular dysfunction to hemodynamic consequences. Intriguingly, multivariate regression identified eNOS and IL-10 expression as independent indicators of endometrial vascular competence. The data suggest that NOS3 polymorphisms may not function independently but instead interact with inflammatory mechanisms to regulate implantation success [22].

A compelling area of research now being explored is the post-transcriptional regulation of eNOS. Jia et al. (2016) identified apolipoprotein A1 (apoA1) and heterogeneous nuclear ribonucleoprotein E1 (hnRNP-E1) as key stabilizers of eNOS mRNA in animal models. Decreased eNOS expression, increased lipid peroxidation, and diminished implantation and birth rates resulted from the knockdown of either protein. Their findings enhance the understanding of endometrial receptivity by associating nitric oxide signaling with chromosomal variability, RNA-binding protein dynamics, and oxidative stress responses. This is significant as it indicates that the dysregulation of RNA metabolism may replicate the same molecular phenotype—specifically, reduced NO output and failed implantation—even in women lacking adverse NOS3 genotypes [59].

Luque et al. (2023) present more evidence for the gene–environment axis, demonstrating in mouse models that hormonal imbalance, particularly involving ghrelin, modifies the expression of both eNOS and iNOS throughout the peri-implantation period. Their research demonstrated that both hyperghrelinemia and ghrelin antagonism induced nitrosative stress, disrupted immunological homeostasis (notably concerning NK/T cell ratios), and diminished embryo implantation. This model robustly indicates that NOS3 function is responsive to systemic hormonal signals, suggesting that these interactions may uncover latent genetic risks or exacerbate current dysfunctions in the NO signaling pathways [60].

Zullino et al. (2018) demonstrated the regulatory role of estrogen on NOS3 transcription and how its levels can either amplify or attenuate the phenotypic consequences of inherent NOS3 mutations. In conditions such as luteal phase failure or endometriosis, characterized by estrogen deficiency or dominance, the compensatory regulation of nitric oxide may be compromised, resulting in abnormal endometrial perfusion, inadequate decidualization, or inappropriate immune activation. This hormonal–genetic synergy is clinically significant, especially for IVF patients facing recurrent implantation failure or inadequate response [39].

### 4.3. Considerations from Experimental Modulation

The intricate role of NO in regulating endometrial receptivity has been significantly enhanced by experimental alterations in the system via pharmacological, genetic, or hormonal manipulations. This collection of papers demonstrates that NO acts as a molecular integrator of vascular, immunological, hormonal, and redox signaling in the endometrium, highlighting that both insufficient and excessive NO production may disrupt implantation. To enhance endometrial perfusion, a notable study by Ohl et al. investigated the application of transdermal nitroglycerin, a nitric oxide donor, in women undergoing in vitro fertilization. The clinical trial, however, did not demonstrate any significant enhancement in implantation or pregnancy rates. This outcome highlights the challenges of systematic nitric oxide supplementation, which is characterized by a lack of tissue selectivity and temporal regulation. Excess nitric oxide can lead to the downregulation of integrin signaling, S-nitrosylation of cellular proteins, and nitrosative stress, all of which adversely affect blastocyst adhesion. This suggests that systemic nitric oxide may activate iNOS pathways in immune cells, thus creating a detrimental microenvironment. Consequently, unregulated nitric oxide supplementation may paradoxically diminish the redox-sensitive implantation window, highlighting the necessity for spatially and temporally targeted strategies [36].

In contrast to therapeutic interventions, in vitro research has yielded a more intricate comprehension of the downstream effects of NO signaling. Melford et al. demonstrated that exogenous NO donors elevated key receptivity indicators, such as HOXA10, LIF, and integrin β3, in a dose-dependent manner in human endometrial epithelial cells. These molecules demonstrate that physiological levels of nitric oxide enhance cellular communication during blastocyst adhesion and stromal–epithelial interaction in the implantation environment. Particularly using FAAH and NAPE-PLD, their research elucidated the intersection of nitric oxide and endocannabinoid metabolism, hence influencing lipid-derived signaling pathways in the endometrium. This indicates that NO functions as a secondary messenger in metabolic networks that regulate epithelial integrity, immunological tolerance, and matrix remodeling, hence extending beyond vasodilation. Moreover, indicating an epigenetic role in cellular differentiation, NO appears to regulate chromatin accessibility at the promoters of decidual genes. These findings validate the research of Su et al., which showed that excessive nitric oxide in endometrial cells leads to mitochondrial dysfunction, cytochrome c release, and caspase-3 activation, thereby inducing apoptosis. These findings collectively underscore the necessity for precisely regulated NO concentrations since both insufficient and excessive amounts result in implantation failure through distinct molecular mechanisms [15].

Preclinical models elucidate the hormonal regulation of nitric oxide. Luque et al. (2023) showed that an imbalance of ghrelin in mice, whether through excess or antagonism, resulted in significant alterations in uterine NOS isoform expression. Elevated levels of both eNOS and iNOS indicated significant nitrosative stress, as evidenced by the accumulation of nitrotyrosine. This imbalance resulted in heightened embryo resorption, reduced implantation success, and altered uterine leukocyte counts, characterized by an increase in dendritic cells and a decrease in T cells. Notably, IL-10 expression was suppressed, whereas pro-inflammatory cytokines such as IL-6 and IL-17 were elevated, suggesting that dysregulated nitric oxide production induces a shift from tolerance to inflammation. These findings integrate the endocrine, immunologic, and redox pathways of implantation, substantiating the hypothesis that nitric oxide regulates both the uterine immune environment and vascular tone [60]. Similarly, Chwalisz et al. illustrated that mifepristone’s inhibition of progesterone receptors diminished eNOS expression and obstructed decidualization, thus indicating that nitric oxide functions downstream of steroid signaling to orchestrate vascular and stromal preparation. The decidual phenotype is primarily influenced by progesterone, making the hormonal–NO interaction essential; therefore, its subsequent effects must be optimally regulated to facilitate embryo tolerance and vascular remodeling [45].

Another significant control point in implantation has been identified as the post-transcriptional modulation of eNOS and nitric oxide bioavailability [61]. The preservation of eNOS expression during the peri-implantation period relies on two mRNA stability regulators, apolipoprotein A1 (ApoA1) and hnRNP-E1 [59]. In mice, the inhibition of either component led to significant reductions in eNOS protein levels, elevated malondialdehyde (MDA, an indicator of lipid peroxidation), and diminished SOD, therefore highlighting the redox consequences of impaired NO generation. This resulted in diminished implantation rates and a lower number of live births [62]. These findings indicate that nitric oxide generation is regulated not only at the genetic level but also significantly associated with oxidative stress regulatory mechanisms and mRNA-binding protein networks. These findings suggest that therapies aimed at stabilizing NOS3 transcripts or enhancing ApoA1 activity may provide a novel strategy to enhance implantation outcomes, especially in individuals with diminished eNOS expression due to environmental or genetic factors.

Additionally, the metabolic restoration of the nitric oxide system demonstrates significant translational importance. Voros et al. (2024) conducted a thorough investigation revealing that bariatric surgery in obese women elevated NO_2_^−^/NO_3_^−^ levels by 45% and simultaneously enhanced pregnancy rates. These alterations were attributed to enhanced mitochondrial activity, reduced oxidative stress, and reinstated nitrate–nitrite–NO conversion, thereby counteracting eNOS dysfunction. The work indicates that, especially during oxidative stress or eNOS uncoupling, there may be an alternative pathway for producing NO that might be pharmacologically or metabolically enhanced. This underscores the absence of “reserve capacity” and illustrates the correlation between endometrial nitric oxide dynamics, reproductive outcomes, and systemic metabolic health [27].

Banerjee et al. demonstrated that women with unexplained recurrent miscarriage exhibited significantly reduced production of eNOS, VEGF, and IL-10 alongside modified subendometrial blood flow, as assessed by Doppler ultrasound, providing more evidence for the vascular role of nitric oxide. This highlights that a deficiency of molecular NO manifests as macroscopic vascular dysfunction in addition to being an intracellular issue [22]. Agarwal et al. similarly showed that nitric oxide shortage elevates lipid peroxidation and disrupts mitochondrial respiration, hence undermining endometrial immune regulation mediated by cytokines [18]. Zullino et al. proposed that dysregulation leads to altered microRNA production in extracellular vesicles, potentially affecting stromal cell differentiation and inflammatory signaling, while Telfer et al. confirmed the necessity of nitric oxide in maintaining endothelial barrier integrity [39].

### 4.4. Integration with Pathophysiological Models

The NO pathway serves as a biochemical link among endothelial function, immune regulation, hormonal signaling, and oxidative stress, and it is increasingly being recognized as a pivotal junction in several gynecological disorders that impair fertility [27]. Dysregulation, whether due to genetic polymorphisms, epigenetic modifications, or abnormal hormonal control, appears to be both a consequence of pathology and a contributing factor to disease progression in conditions such as endometriosis, PCOS, obesity-related subfertility, and IRPL [63]. The comprehensive data indicates that nitric oxide’s role in angiogenesis, decidualization, immunological tolerance, and cellular redox homeostasis is essential for the formation of a receptive endometrium; any disruption in these processes results in implantation failure and early pregnancy loss.

A quintessential example of this integrated function is endometriosis. Chronic eNOS overexpression in the eutopic endometrium was associated with vascular disorganization and impaired tissue remodeling in women experiencing infertility due to endometriosis [64]. The abnormalities were reversed, particularly with GnRH agonist treatment, indicating that NO functions as a hormonally regulated effector of endometrial repair. Loizidou et al. investigated the association between chronic inflammatory disorders of the uterus, oxidative damage, and elevated iNOS activity, resulting in hypervascularization, stromal fibrosis, and altered paracrine signaling. These findings corroborate the results of Su et al., who indicated that excessive nitric oxide disrupts mitochondrial function and activates caspase-3-mediated apoptosis, hence exacerbating endometrial injury [32].

Banerjee et al. performed a comprehensive case–control study involving 66 women with IRPL within the context of recurrent pregnancy loss, demonstrating that diminished levels of eNOS, VEGF, and IL-10 in the endometrium were significantly associated with compromised subendometrial blood flow, as assessed by color Doppler ultrasonography. Their multivariate analysis confirmed eNOS and IL-10 as independent indicators of vascular dysfunction [22]. Makino et al. observed elevated systemic NO_2_^−^/NO_3_^−^ levels without significant variations in tissue-level eNOS, proposing that systemic oxidative signaling may contribute to uterine endothelial dysfunction through circulating immune cells or nitric oxide synthase uncoupling. Agarwal et al. revealed that redox imbalance, elevated peroxynitrite levels, and lipid peroxidation diminish endometrial receptivity by disrupting cytokine signaling and tissue remodeling capabilities, which is also evident in their data [25].

In obesity and metabolic syndrome, nitric oxide imbalance appears to be both a contributor to reproductive dysfunction and a consequence thereof. Post-bariatric surgery, women exhibited a 45% elevation in nitrate–nitrite levels and a 60% enhancement in conception rates, as reported in a thorough study by Voros et al. (2024) [27]. Accompanying these effects were reduced oxidative stress, enhanced mitochondrial biogenesis, and increased endometrial receptivity. The findings emphasize that under settings of persistent oxidative stress and eNOS uncoupling—common in severe obesity—the alternative nitrate–nitrite–NO pathway functions as a compensatory mechanism to preserve vascular and endocrine equilibrium. This finding indicates that metabolic stiffness at the vascular level disrupts cyclic nitric oxide regulation, consistent with prior research by Battaglia et al. and Fabregues et al., which documented diminished nitric oxide variations during the menstrual cycle in women with suboptimal implantation [26,46].

Furthermore, nitric oxide widely permeates the connection among immunological, endocrine, and endothelial systems. In a murine model, Luque et al. showed that ghrelin dysregulation—through either agonism or antagonism—resulted in elevated uterine iNOS and eNOS synthesis, thereby causing nitrotyrosine accumulation and leukocyte infiltration. Concomitantly, there was a decrease in regulatory cytokines such as IL-10, with an increase in the production of IL-6, IL-17, and MMP-9. Significantly, T cell counts decreased, while NK and dendritic cell populations increased, indicating a transition from immunological tolerance to inflammation. These findings augment those of Chwalisz et al., who demonstrated that the inhibition of progesterone receptors reduces eNOS and disrupts decidualization [45]. Collectively, these investigations demonstrate that successful implantation necessitates the hormonal synchronization of nitric oxide levels. Both excess and deficiency precipitate a cascade of immune-mediated rejection of the embryo, especially within a pro-inflammatory milieu.

The function of nitric oxide acquires an additional dimension when integrated into extracellular signaling networks and epigenetic pathways. No, Zullino et al. indicated that alterations in the contents of extracellular vesicles—including microRNAs (miR-21 and miR-200b)—regulate gene expression in recipient stromal and immune cells. Angiogenesis, matrix remodeling, and immune surveillance rely on this form of paracrine communication [39]. Andronico et al. and Muraoka et al. further emphasized the impact of oxidative stress and nitric oxide imbalance on trophoblast-derived extracellular vesicles, hence disrupting embryo–endometrium contact. These findings designate NO as a transcellular coordinator of reproductive competence, thus broadening its role beyond intracellular signaling [40,41].

Impaired NO signaling may restrict receptivity despite the presence of a structurally normal endometrium. Najafi et al. and Banerjee et al. demonstrated that eNOS overexpression, despite the absence of discernible histological defects, might alter adhesion molecule expression, integrin distribution, and glycocalyx integrity, thereby compromising the endometrium’s receptivity at the molecular level. The combination of findings, ranging from DNA polymorphisms to mRNA stability and protein activity, with data by Jia et al., which demonstrated the post-transcriptional instability of eNOS through ApoA1 or hnRNP-E1 knockdown, substantiates the regulation of nitric oxide at all hierarchical levels of gene expression [16,22].

### 4.5. Translational Prospects and Clinical Implications

Clinical practice in reproductive medicine will undergo significant transformation if NO is recognized as a crucial regulatory component in the molecular framework of endometrial receptivity. Nitric oxide is increasingly being recognized as a principal regulator of various processes pertinent to implantation, encompassing hormone responsiveness, immunological tolerance, oxidative equilibrium, extracellular matrix remodeling, and angiogenesis, rather than merely serving as a vascular modulator. In cases of unexplained infertility, RIF, and RPL, its dual role as both a biomarker and a therapeutic target offers significant translational potential for the diagnosis and treatment of infertility. Nitric oxide has distinctive molecular plasticity: at physiological concentrations, it facilitates uterine vascularization, stromal decidualization, and immunological quiescence. Conversely, at pathological concentrations—either insufficient or excessive—it induces endothelial dysfunction, nitrosative stress, and unsuccessful implantation. Clinical therapies must therefore be grounded in precision-based frameworks that account for a patient’s nitric oxide profile across hormonal, genetic, metabolic, and immunologic dimensions.

The development of biomarkers is a valuable application. Numerous studies, including those by Makino et al., Roychoudhury et al., Banerjee et al., and Fabregues et al., demonstrate that peripheral levels of NO_2_^−^ and NO_3_^−^ metabolites differ between women who achieve conception and those who do not. While the correlation between serum NO metabolites and intra-endometrial NO bioactivity remains imperfect, our findings suggest that systemic markers may serve as surrogate indicators of vascular and redox status. To enhance diagnostic precision, a multi-analyte panel encompassing oxidative stress markers (e.g., MDA, 8-OHdG, and SOD), inflammatory cytokines (e.g., IL-10 and TNF-α), and hormonal factors (e.g., estradiol and progesterone) should be considered, notwithstanding the potential lack of correlation between circulating NO metabolites and localized uterine phenomena [22,25,26,33]. Furthermore, advancements in liquid biopsy technologies—specifically those quantifying microRNAs in extracellular vesicles (Zullino et al. and Muraoka et al.)—present the potential for the non-invasive surveillance of implantation-associated molecular markers, many of which are directly affected by nitric oxide levels. Utilizing a “redox receptivity index” based on these criteria, reproductive endocrinologists could classify patients into functionally hypo-NO, normo-NO, or hyper-NO phenotypes, thereby directing therapy with unprecedented precision [39,41].

From a genetic perspective, NOS3 polymorphisms provide an additional layer of clinical importance. Hefler et al., Karvela et al., and Budani et al. identified variations such as Glu298Asp and the intron 4 VNTR to be associated with an increased risk of miscarriage and suboptimal IVF outcomes. These SNPs either alter enzyme structure, reduce dimer stability, or disrupt mRNA expression, hence affecting NO production. In women with idiopathic subfertility or a history of recurrent implantation failure, incorporating NOS3 genotyping into preconception screening might facilitate genotype-based classification for treatment options. Women possessing low-activity NOS3 genotypes may benefit from tailored L-arginine or citrulline supplementation, which enhances nitric oxide synthesis, or the concurrent administration of BH_4_ (tetrahydrobiopterin), a crucial cofactor for eNOS coupling [23,38]. Moreover, the molecular models demonstrated by Agarwal et al. and Su et al. indicate that antioxidants such as vitamins C and E may inhibit eNOS uncoupling in genetically predisposed people. These findings endorse a functional nutritional strategy in which food and supplements are tailored according to NO-related genotypes to optimize reproductive capacity [52,65].

Therapeutically, the direct modification of nitric oxide bioavailability offers intriguing although complex potential. Initial experiments conducted by Ohl et al. utilizing transdermal nitroglycerin to enhance uterine perfusion revealed the constraints of systemic nitric oxide availability. The generic nature of transdermal nitric oxide release likely resulted in off-target effects, including potential systemic hypotension and paradoxical nitrosative stress, which is conceptually intriguing. These results necessitate more sophisticated methodologies. Targeted liposomal nanoparticles, biodegradable polymer microspheres, or intrauterine gels—localized nitric oxide-releasing devices—may offer precise spatial and temporal regulation of nitric oxide distribution [36]. These devices would enable physicians to duplicate natural nitric oxide pulsatility during the implantation period, thereby enhancing tissue-specific effects on immunological tolerance, stromal differentiation, and vascular permeability without causing systemic side effects. Future nitric oxide-based therapies may additionally integrate PDE5 inhibitors or cGMP analogs, which enhance nitric oxide signaling downstream by inhibiting cGMP degradation. Due to their titrated and localized effects, these agents—currently employed in cardiovascular and erectile dysfunction contexts—may be applicable in IVF procedures involving NO-deficient endometrial profiles.

Emerging research on the post-transcriptional control of eNOS introduces a novel pharmacologic pathway. Jia et al. demonstrated that during the peri-implantation phase, RNA-binding proteins such as ApoA1 and hnRNP-E1 stabilize NOS3 transcripts and enhance the production of NO. The therapeutic targeting of these proteins—through small-molecule agonists or mRNA stabilizing agents—may assist women with unexplained nitric oxide deficiency in maintaining eNOS expression. Therapies enhancing mitochondrial integrity, such as coenzyme Q10, resveratrol, or PGC-1α activators, may reduce ROS production and safeguard eNOS functionality by maintaining the requisite redox state for enzymatic activity [59]. According to Voros et al. (2024) and Luque et al., such therapies may correct upstream metabolic disturbances and restore nitric oxide homeostasis in women with obesity or polycystic ovary syndrome, who generally exhibit redox imbalance, hormonal dysregulation, and endothelial dysfunction [27,60].

The utilization of NO-based diagnostics and therapeutics in the optimization of ART represents yet another compelling area of translation. Assessing follicular fluid nitric oxide levels post-oocyte extraction may serve as an indicator of systemic redox equilibrium; increased peroxynitrite indicates a necessity for antioxidant preconditioning [66]. Furthermore, real-time evaluation of uterine nitric oxide flux using biosensors or molecular imaging technologies may facilitate the timing of embryo transfer during a patient’s optimal receptivity period [67]. Incorporating these nitric oxide-related diagnostics into IVF techniques will reduce embryo wastage and enhance implantation precision. Customized NO modulation may be integrated with conventional ovarian stimulation and luteal support methods in forthcoming treatments. Hypo-NO patients may benefit from localized NO donors or activators of upstream pathways, whereas women with hyper-NO profiles could utilize selective iNOS inhibitors or anti-inflammatory cytokine therapy to reduce excess NO and enhance immunological tolerance.

The therapeutic application of nitric oxide studies in reproduction will necessitate a multimodal, interdisciplinary strategy. This discipline requires expertise in vascular biology, immunology, pharmacology, and systems biology, in addition to gynecology and reproductive endocrinology. Distinct nitric oxide networks can be mapped and patient-specific receptivity profiles can be developed by integrating multi-omics methodologies—genomics, transcriptomics, proteomics, and metabolomics. By integrating genetic variants (NOS3 and VEGF), biochemical markers (NO_2_^−^, NO_3_^−^, and MDA), hormonal profiles, and the uterine immunological environment, future reproductive medicine may offer a nitric oxide “dashboard” for each patient to recommend personalized therapy. These may encompass dietary strategies, antioxidant combinations, mRNA therapies, and endometrial-targeted nitric oxide administration. In this context, NO emerges not only as a focal point of interest but also as a crucial therapeutic axis, enabling the personalization of IVF and other reproductive treatments to an unprecedented extent.

### 4.6. Constraints and Prospective Avenues

This systematic review provides a comprehensive synthesis of the role of NO, its metabolites, and eNOS activity in endometrial receptivity and implantation; nevertheless, certain methodological and conceptual limitations must be acknowledged. The included research exhibits considerable variability in design, sample size, timing, and analytical methodologies. Researchers employed several methodologies to quantify nitric oxide, including serum nitrate/nitrite measurements (Makino et al. and Roychoudhury et al.) [25,33], eNOS immunohistochemistry (Najafi et al. and Banerjee et al.) [16,22], and gene polymorphism analysis (Hefler et al. and Karvela et al.) [23,24], hence allowing for direct comparisons and data compilation. Furthermore, limited research has simultaneously evaluated systemic nitric oxide metabolites alongside local endometrial expression, hence constraining our ability to correlate tissue-level function with peripheral markers. Moreover, certain studies incorporated confounders like BMI, hormone levels, or cycle phases, while others excluded them, thereby raising concerns over unmeasured bias and population-specific effects. Research on nitric oxide signaling in polycystic ovary syndrome or obesity is notably underrepresented despite the acknowledged connection between nitric oxide and metabolic illnesses.

Secondly, numerous clinical investigations employed molecular approaches characterized by inadequate sensitivity and specificity to investigate nitric oxide dynamics. Colorimetric Griess assays for NO_2_^−^/NO_3_^−^ are prevalent; however, they fail to distinguish between NO produced by eNOS and that generated by iNOS during inflammatory processes. The enzymatic source of NO is crucial as it dictates its biological effects: iNOS-derived NO induces nitrosative stress and apoptosis, whereas eNOS-derived NO facilitates vasodilation and trophoblast invasion. Limited research has differentiated these isoforms at the mRNA or protein level (e.g., Luque et al. and Chwalisz et al.) [45,60], and even fewer have examined the dynamic equilibrium between them. The absence of time-course data on nitric oxide changes during the implantation window in numerous experimental models may elucidate the optimal timing for therapeutic intervention. Despite encouraging in vitro studies (Melford et al. and Jia et al.) demonstrating downstream NO-sensitive genes and signaling pathways, their use in clinical practice remains uncertain due to the lack of longitudinal and interventional research [15,59].

The integration of the hormonal and immunological axes presents an additional critical issue. Despite robust data demonstrating that estrogen signaling and progesterone meticulously regulate nitric oxide, limited research has specifically connected blood hormone levels with endothelial nitric oxide synthase production or activity. This association constrains our understanding of how hormonal dysregulation, commonly observed in conditions such as luteal phase insufficiency or endometriosis, translates into altered nitric oxide bioavailability. The immunological profile reflects a similar situation: whereas studies by Luque et al. demonstrated significant NO-dependent alterations in uterine leukocyte counts, human trials have not corroborated these findings. The role of NO-mediated signaling in the uterine immune milieu, particularly for the roles of uNK cells, dendritic cells, and regulatory T cells, is little explored [60]. Although these molecular layers have not yet been integrated into significant clinical research or diagnostics, emerging fields like as microRNA and extracellular vesicle biology (Zullino et al. and Muraoka et al.) offer compelling evidence for NO-mediated paracrine regulation [39,41].

Future investigations should prioritize multi-layered, longitudinal, and translational studies. Initially, robust, prospective clinical trials are necessary to examine the effectiveness of nitric oxide-modulating therapies—including dietary nitrate supplementation, specific nitric oxide donors, and mitochondrial-supportive agents—in improving implantation rates. Highlighting a personalized medicine strategy, these medicines ought to be classified according to the NOS3 genotype, metabolic phenotype (e.g., BMI and insulin resistance), and redox status. Secondly, high-resolution omics technologies such as single-cell RNA sequencing, proteomics, and spatial transcriptomics should be employed to delineate NO-dependent cellular networks throughout the endometrium throughout the secretory phase. Distinguishing among epithelial, stromal, vascular, and immune compartments facilitates the identification of the spatial specificity of NO signaling and the localization of molecular switches governing the receptacle. The incorporation of artificial intelligence techniques for multi-omics data fusion may enhance the development of predictive models linking nitric oxide dynamics to in vitro fertilization outcomes.

Furthermore, subsequent studies must enhance the temporal resolution of NO profiling. The repeated collection of endometrial biopsies or uterine fluid during the peri-implantation period may elucidate whether existing therapies (e.g., progesterone support, GnRH analogs, and metformin) restore these cycles and how nitric oxide levels differ between fertile and infertile individuals. The simultaneous advancement of non-invasive biosensors or NO-responsive imaging tracers may enable a real-time assessment of uterine receptivity in IVF cycles. Moreover, it is warranted to incorporate epigenetic regulation—encompassing NO-mediated alterations in histone modifications and DNA methylation—into the NO research framework. Jia et al. demonstrate that the post-transcriptional manipulation of redox-sensitive mRNA regulators and NOS3 can substantially affect implantation potential. Understanding the interaction between environmental challenges (such as inflammation, obesity, and endocrine disruptors) and epigenetic checkpoints may lead to novel therapies aimed at restoring nitric oxide signaling in compromised endometria [59].

Ultimately, standardized nitric oxide measurement methodologies, improved repeatability, and the convenience of meta-analysis will necessitate global collaboration. These endeavors could establish reference values for nitric oxide metabolites and endothelial nitric oxide synthase activity in fertile women, against which patients with infertility can be compared. Incorporating NO-specific modules into current fertility biobanks or IVF registries will expedite this process and facilitate the real-world validation of proposed NO-based therapies. Integrating molecular biology, clinical endocrinology, systems medicine, and reproductive immunology is essential for the comprehensive translation of nitric oxide signals as these signals are both genetically determined and influenced by environmental factors.

## 5. Conclusions

This thorough investigation emphasizes the vital role of NO and its related molecular pathways—particularly eNOS activity and the nitrate–nitrite–NO axis—in the organization of the endometrial receptacle and the successful implantation of embryos. Nitric oxide serves as a principal regulator of critical reproductive processes, encompassing decidualization, angiogenesis, immunological tolerance, and redox homeostasis across many clinical contexts and experimental frameworks. Insufficient NO diminishes vascular remodeling, leukocyte recruitment, and endometrial differentiation; yet the reviewed data indicates that both elevated and diminished NO levels can be harmful: supraphysiological NO induces nitrosative stress, mitochondrial malfunction, and apoptotic signaling. This duality underscores the necessity of meticulously managed nitric oxide balance during the implantation phase, both spatially and temporally.

The eNOS function and subsequent NO bioavailability can be significantly affected by genetic predispositions, including NOS3 polymorphisms such as Glu298Asp and intron 4 VNTR. Hormonal cues, metabolic conditions, the inflammatory environment, and post-transcriptional regulators all influence these variants. Systemic illnesses such as endometriosis, insulin resistance, and obesity exacerbate nitric oxide dysregulation through oxidative stress and hormonal imbalance, hence elevating the likelihood of implantation failure. The findings from animal and human studies validate that the nitrate–nitrite–NO pathway operates as a compensatory mechanism in hypoxic or oxidative environments, partially restoring NO signaling in cases of eNOS dysfunction.

These discoveries possess significant translational potential. Numerous potential avenues for patient classification and therapeutic targeting exist through peripheral biomarkers (NO_2_^−^, NO_3_^−^, and oxidative stress indicators), the endometrial expression of eNOS, NOS3 genotyping, and the advancement of microRNA or extracellular vesicle profiling. Despite the inconsistent results of comprehensive clinical trials involving NO donors, the subsequent phase in personalized reproductive medicine involves the creation of localized, timed-release NO delivery devices and therapies aimed at enhancing endogenous NO production. Furthermore, interventions targeting the stabilization of NOS3 mRNA, mitochondrial function, and immune modulation may expand the resources available for optimizing endometrial receptivity.

Ultimately, the essence of implantation biology is a dynamic and multi-faceted regulatory axis of nitric oxide signaling. In women with unexplained infertility or inadequate responses to assisted reproduction, optimizing this pathway—via genetic diagnoses, tailored medications, and integrated omics approaches—may enhance reproductive outcomes. Future studies must prioritize translational models that integrate genetic, hormonal, metabolic, and immunologic aspects to fully harness the diagnostic and therapeutic potential of nitric oxide in human fertility.

## Figures and Tables

**Table 1:** Eligibility criteria based on the PICOS framework.

PICOS Element	Description
**Population (P)**	Women of reproductive age undergoing IVF and women with UI, RPL, or RIF. Studies involving healthy fertile controls for comparison were also included.
**Intervention/Exposure (I)**	The evaluation or modulation of NO, its metabolites (nitrite/nitrate), or eNOS expression. This includes molecular, biochemical, genetic, and clinical assessments.
**Comparison (C)**	Fertile women, successful IVF cycles, or normal endometrial receptivity were used as controls for comparison with infertile populations or failed IVF outcomes.
**Outcomes (O)**	Primary outcomes included the implantation rate, clinical pregnancy, miscarriage, expression levels of NO/eNOS, the presence of NOS3 gene polymorphisms, and concentrations of NO_2_^−^/NO_3_^−^.
**Study Design (S)**	Eligible designs included case–control studies, prospective or retrospective cohort studies, randomized controlled trials (RCTs), and mechanistic in vitro studies using human endometrial tissue or cell lines. Reviews, editorials, case reports, and animal studies were excluded.

This table delineates the established inclusion and exclusion criteria employed to identify studies for this systematic review, structured according to the Population, Intervention/Exposure, Comparison, Outcomes, and Study design (PICOS) framework.

**Table 2:** Search strategies used in PubMed, Scopus, and Web of Science.

Database	Search Strategy	Filters Applied
PubMed	(“nitric oxide”[MeSH Terms] OR “nitric oxide”[Title/Abstract] OR “NO”[Title/Abstract] OR “eNOS”[Title/Abstract] OR “NOS3”[Title/Abstract] OR “nitrate”[Title/Abstract] OR “nitrite”[Title/Abstract]) AND (“endometrium”[MeSH Terms] OR “endometrial receptivity”[Title/Abstract] OR “implantation”[MeSH Terms] OR “implantation”[Title/Abstract]) AND (“in vitro fertilization”[MeSH Terms] OR “IVF”[Title/Abstract] OR “assisted reproduction”[Title/Abstract])	Humans, English, Full Text
Scopus	TITLE-ABS-KEY (“nitric oxide” OR “NO” OR “eNOS” OR “NOS3” OR “nitrate” OR “nitrite”) AND TITLE-ABS-KEY (“endometrium” OR “endometrial receptivity” OR “implantation”) AND TITLE-ABS-KEY (“IVF” OR “in vitro fertilization” OR “assisted reproduction”)	Article type: Research Article; Language: English
Web of Science	TS = (“nitric oxide” OR “eNOS” OR “NOS3” OR “nitrate” OR “nitrite”) AND TS = (“endometrium” OR “implantation” OR “endometrial receptivity”) AND TS = (“IVF” OR “in vitro fertilization” OR “assisted reproduction”)	Document Type: Article; Language: English

Table 2 lists the particular search phrases and Boolean operators used in several databases to find qualified research about nitric oxide (NO), eNOS, and its relationship with endometrial receptivity and IVF results. All searches were performed on 31 March 2024.

**Table 3:** Evaluation of bias risk in included studies based on study design.

Author (Year)	Study Design	Assessment Tool	Risk of Bias Judgment	Comments
Al Sallout (2010) [31]	Case–control	NOS	Moderate (6/9)	Clear definition of cases/controls; no adjustment for confounders
Banerjee (2013) [22]	Case–control	NOS	High (4/9)	Limited reporting on selection methods and comparability
Fábregues (2000) [26]	Prospective cohort	NOS	Moderate (6/9)	Large sample; no clear control for confounders
Hefler (2002) [24]	Case–control	NOS	Moderate (6/9)	Good matching; lack of detailed exposure validation
Karvela (2008) [23]	Case–control	NOS	Low (7/9)	Well-matched groups; adequate reporting
Loizidou (2021) [32]	Case–control	NOS	Low (7/9)	Strong design and sample characterization
Makino (2004) [25]	Case–control	NOS	Moderate (6/9)	Valid exposure measurement; no power calculation
Najafi (2012) [20]	Case–control	NOS	High (4/9)	Small sample size; unclear exposure ascertainment
Najafi (2013) [16]	Case–control	NOS	Moderate (6/9)	Improved sample definition; no confounder adjustment
Roychoudhury (2016) [33]	Case–control	NOS	Moderate (6/9)	Case/control definition clear; some selection bias possible
Wang (2006) [34]	Prospective cohort	NOS	Low (7/9)	Robust measurement; hormonal correlation supported
Sun (2003) [35]	Prospective cohort	NOS	Moderate (6/9)	Small sample; outcome measurement valid
Ohl (2002) [36]	RCT	RoB 2.0	Some concerns	Possible bias in outcome measurement and selective reporting
Melford (2021) [15]	In vitro	Narrative appraisal	Moderate	Well-controlled but not externally validated across systems

This table displays the assessment of methodological quality for the included papers, utilizing the Newcastle–Ottawa Scale (NOS) for observational research, the Cochrane Risk of Bias 2.0 tool for randomized controlled trials, and narrative criteria for in vitro investigations. The risk levels are classified as low, moderate, or high, accompanied by remarks on certain limitations or strengths.

**Table 4:** Methodological features of included studies evaluating NO, NO_2_-NO_3_, or eNOS in relation to endometrial receptivity and IVF outcomes.

Year	Author	Type of Study	Inclusion Criteria	Exclusion Criteria	Recruitment Period	Sample size (Cases/Controls)	Assessment Performed	Results
2010	Al Sallout et al. [31]	Case–control study	Cases Women who had experienced at least 3 unexplained consecutive spontaneous miscarriages prior to 25 weeks of gestation Diagnosis of idiopathic recurrent miscarriages Controls Proven fertile women undergoing menopause No use of oral contraceptives, hormonal, or intrauterine devices No use of any medication affecting liver function and/or blood coagulation	Negative for Toxoplasma IgM, CMV IgM, Chlamydia IgM, Rubella IgM, ACL IgG or IgM, and APL IgG or IgM	2007–2008	100/100	Polymorphisms in NOS3 genes	Intron 4 (4a/4b) polymorphism of NOS3 gene is not significantly associated with RM 4a/4a genotype appeared increased in RM but non-significantly
2013	Banerjee et al. [22]	Prospective case–control study	Cases Women who had experienced at least 3 unexplained consecutive spontaneous miscarriages prior to 12 weeks of gestation <35 years old BMI ≤ 28 Indian origin Controls Proven fertile women undergoing sterilization No history of failed pregnancies No significant clinical abnormalities	Thyroid disorders APS Infectious diseases Paternal or maternal chromosomal abnormalities Uterine defects PCOS DM Luteal phase defect Thrombophilia Hyperhomocystinemia	Not mentioned	66/50	eNOS Plasma NO levels	eNOS and NO levels were significantly lower in cases during implantation window
2000	Fábregues et al. [26]	Prospective cohort	Women attending infertility clinic that underwent IVF or ET	Unilateral or bilateral adnexectomy Smoking Intense exercise	Not mentioned	237	NO_3_-NO_2_ on days 13–14 and 20–21 after ET	No significant differences in serum nitrite/nitrate in conception versus non-conception cycles, viable versus abnormal pregnancies, or viable pregnancy group versus non-conception cycles
2002	Hefler et al. [24]	Prospective case–control study	Cases Women who had experienced at least 3 unexplained consecutive spontaneous miscarriages prior to 20 weeks of gestation Diagnosis of idiopathic recurrent miscarriages Women and their partners were of white origin Controls Proven fertile women with at least two live births No history of miscarriage Women and their partners were of white origin	Paternal or maternal chromosomal abnormalities Uterine defects	Not mentioned	126/130	Alterations in intron 4 repeat polymorphism or Glu298Asp missense mutation both encoded by eNOS	No significant difference between two groups for either polymorphism
2021	Loizidou et al. [6]	Case–control study	Cases Women who had experienced at least 2 unexplained consecutive spontaneous miscarriages prior to 14 weeks of gestation Controls Proven fertile women with at least one live birth No history of miscarriage or fetal loss	Family history of birth defects Genital tract anatomic abnormalities Paternal or maternal chromosomal abnormalities Hyperprolactinemia APS Infectious diseases DM Thyroid disorders	Not mentioned	114/106	Alterations in intron 4 repeat polymorphism encoded by eNOS	No significant difference between two groups
2004	Makino et al. [25]	Case–control study	Cases Women who had experienced at least 2 unexplained consecutive spontaneous miscarriages prior to 14 weeks of gestation (embryonal loss group) Women with history of at least 1 unexplained mid-trimester or third trimester IUFD and/or FGR (fetal loss group) Controls Proven fertile women with or without live birth No history of miscarriage or fetal loss	Paternal or maternal chromosomal abnormalities APS Infectious diseases DM Thyroid disorders Hyperprolactinemia Genital tract anatomic abnormalities	2000–2003	125/76	Alterations in intron 4 repeat polymorphism encoded by eNOS Plasma NO levels	No significant differences in eNOS alleles between groups Plasma NO levels were significantly increased in study groups compared to control group
2021	Melford et al. [15]	In vivo and in vitro experiments	In vivo component Endometrial biopsies taken throughout menstrual cycle In vitro component Simulation of “receptive” and “non-receptive” endometrium by using Ishikawa and HEC-1A cells, respectively	Not applicable	Not mentioned	Not applicable	In vivo expression of ECS enzymes (FAAH and NAPE-PLD) FAAH and NAPE-PLD expression in “receptive” and “non-receptive” human endometrial cell lines treated with NO-donating compound SNAP	SNAP resulted in increase in amount of *FAAH* mRNA produced by “receptive” cells and decrease in *NAPE-PLD* mRNA No effect of SNAP was observed in non-receptive cells
2005	Wang et al. [34]	Prospective case–control study	Cases Women with endometriosis and primary or secondary infertility who were treated with operative laparoscopy and endometrial biopsy. 18 women postoperatively received GnRH-analogs and underwent controlled ovarian stimulation and endometrial biopsy was collected again in the same stage of the menstrual cycle as the first biopsy Controls Patients with in situ carcinoma of the cervix that underwent endometrial biopsy at the time of hysterectomy of normal eutopic endometrium	Endocrine disorders Medications that could disturb hypothalamo-pituitary– ovarian axis Use of immunosuppressive medications >40 years old	2003–2004	30/19	eNOS and iNOS protein relative levels and serum concentrations of estradiol or progesterone	Positive correlation between serum estradiol or progesterone levels and eNOS protein levels Maximal expression of eNOS in peri-implantation phase of cycle Eutopic endometrium in endometriosis-associated infertility cases before GnRH-agonist treatment showed higher levels of eNOS than that of control group After 3 months of GnRH-agonist therapy, eNOS levels appeared reduced
2012	Najafi et al. [20]	Case–control study	Cases Women with unexplained infertility Controls Women undergoing total hysterectomy with normal menstrual regularity and at least one live birth	Endocrine disorders APS Genital tract anatomic abnormalities Paternal or maternal chromosomal abnormalities	Not mentioned	10/8	Expression of eNOS in endometrial tissue of women with unexplained infertility	eNOS levels in luminal epithelium was increased in women with unexplained infertility compared to fertile women No statistical significant changes in glandular or vascular epithelium
2013	Najafi et al. [16]	Case–control study	Cases Women with unexplained infertility Women with recurrent miscarriages Controls Women undergoing tubal sterilization with normal menstrual regularity and at least one live birth No prior history of pregnancy losses No prior use of assisted reproductive techniques	Women with secondary miscarriages or less than three miscarriages Endocrine disorders APS Genital tract anatomic abnormalities Paternal or maternal chromosomal abnormalities	Not mentioned	20/10	eNOS protein and mRNA levels in endometrium	Increased expression of eNOS in glandular and luminal epithelium of endometrium in women with recurrent miscarriages and unexplained infertility
2002	Ohl et al. [36]	Prospective, double-blind, randomized, placebo-controlled trial	Patients with a history of two or more previous implantation failures despite the transfer two or more embryos of good quality Patients underwent controlled ovarian stimulation was with long agonist protocol combined with recombinant FSH Embryo transfer on day 2 or 3 after oocyte retrieval Presence of at least two embryos of good quality	Hypersensitivity to nitric oxide donors Heart failure Severe anemia High intracranial blood pressure High intraocular blood pressure	1998–2000	138	Efficacy of NO donor (nitroglycerin) on ovarian response, implantation rate, and pregnancy rate when administered day before embryo transfer	No statistically significant difference was observed
2016	RoyChoudhury et al. [33]	Case–control study	Cases (RIF) Women with history of implantation failure from at least three consecutive IVF attempts despite the transfer of 2–3 embryos of high-grade quality Controls (RIS) Women with successful implantation after IVF-ET	>40 years old BMI > 28 Concomitant gynecological disorders Use of medications over the past 3 months	2004–2014	28/24	Serum eNOS levels	eNOS was significantly lower in women with RIF when compared with RIS
2003	Sun et al. [35]	Prospective cohort	Women with proven fertility who received a single dose of 200 mg of mifepristone on Day 2 and underwent endometrial biopsy on Days 6 to 8	No use of hormonal contraceptives or presence of intrauterine device 3 months prior to study No concomitant medication	Not mentioned	9	eNOS expression of endometrium	eNOS was detected in vascular endothelium and glandular epithelium of endometrium Mifepristone significantly decreased eNOS expression in endometrial glandular epithelium but did not affect endothelial eNOS

The key features of the fourteen studies included in this systematic review are compiled in this table. It provides details on the study design, diagnostic groups, inclusion/exclusion criteria, sample sizes, kinds of biological materials evaluated, techniques applied to measure nitric oxide signaling or eNOS expression, and the main clinical or molecular results pertinent to implantation or assisted reproduction.
